# Synthetic lethality in cancer therapy: Mechanisms, models and clinical translation for overcoming therapeutic resistance

**DOI:** 10.1002/ctm2.70586

**Published:** 2026-01-15

**Authors:** Junyan Li, Liyuan Zhang, Yan Shang, Juan Liu, Hailong Zhao

**Affiliations:** ^1^ Department of Medical Genetics Zunyi Medical University Zunyi Guizhou China; ^2^ Key Laboratory of Cell Engineering of Guizhou Province Affiliated Hospital of Zunyi Medical University Zunyi Guizhou China; ^3^ Department of Pathophysiology Zunyi Medical University Zunyi China; ^4^ Guizhou Biomanufacturing Laboratory Affiliated Hospital of Zunyi Medical University Zunyi Guizhou China

**Keywords:** cancer therapeutic resistance, clinical translation, DNA damage response reprogramming, genome‐scale CRISPR screening, synthetic lethality

## Abstract

**Background and Rationale:**

Synthetic lethality (SL)‐based strategies hold significant promise for overcoming therapeutic resistance, a critical bottleneck in cancer treatment where cancer cells evade anticancer therapies, leading to diminished efficacy or treatment failure. The core of SL lies in exploiting tumour‐specific vulnerabilities: drug‐resistant cells often acquire unique genetic defects or compensatory adaptive responses, and SL strategies selectively target genes or pathways dependent on these vulnerabilities to induce specific cell death, thereby reversing resistance.

**Content and Focus:**

This review systematically elaborates on SL mechanisms and the multi‐faceted nature of tumour drug resistance, then focuses on how SL counteracts resistant phenotypes by leveraging resistant cells’ vulnerabilities. We further delineate SL applications in preclinical resistance models, highlight representative SL‐related drugs and predictive biomarkers and critically analyse challenges in clinical translation.

**Conclusion:**

By integrating mechanistic insights, preclinical validation and translational perspectives, this review aims to provide novel insights for precision therapy and a foundational reference to advance SL strategies in overcoming tumour resistance and facilitating their clinical implementation.

**Key points:**

- SL‐based strategies exploit tumour‐specific vulnerabilities in drug‐resistant cells to induce selective cell death and overcome therapeutic resistance.
- This review dissects SL mechanisms, diverse drivers of tumour drug resistance and how SL counteracts resistant phenotypes via these vulnerabilities.
- It summarises clinical translational applications of SL from preclinical studies to trials, approvals and emerging targets, and discusses future precision therapy.

## Graphical abstract

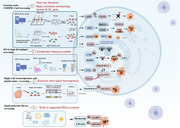

Synthetic lethality (SL) is defined as a genetic interaction wherein concurrent functional loss of two genes results in cellular or organismal death, while inactivation of either gene alone remains compatible with survival.^1^ This unique biological phenomenon has emerged as a transformative paradigm in oncology, primarily owing to its capacity to address three critical limitations of conventional cancer therapies: reversing acquired or intrinsic drug resistance, minimising off‐target toxicity and enhancing the precision of therapeutic targeting. These attributes position SL as a promising strategy to overcome the bottlenecks in current cancer treatment modalities. A paradigmatic illustration of SL‐mediated targeted therapy resides in the DNA damage repair pathway: inhibitors of poly (ADP‐ribose) polymerase (PARP), encoded by PARP1, exploit SL to selectively eliminate cancer cells harbouring deficiencies in BRCA1 or BRCA2.^2^ By capitalising on the interdependence between PARP‐mediated single‐strand break (SSB) repair and BRCA‐dependent homologous recombination (HR), PARP inhibitors (PARPis) induce irreparable genomic damage specifically in BRCA‐deficient cells, thereby achieving selective cytotoxicity.

Tumour drug resistance, a pivotal obstacle in clinical oncology, denotes the adaptive capacity of cancer cells to evade the cytotoxic effects of anti‐cancer therapies post‐treatment, culminating in diminished therapeutic efficacy or outright treatment failure. This multi‐faceted process is orchestrated by a complex interplay of genetic and signalling pathway perturbations, encompassing heightened drug efflux mediated by transporters such as ABCB1 (P‐gp),^3^, ^4^ impaired drug uptake via solute carrier transporters,^5^ augmented DNA damage repair machinery^6^ and dysregulated apoptotic signalling,^7^ among other mechanisms. Clinically, resistance manifests as two primary phenotypes: primary non‐response, observed in approximately 20–30% of patients receiving first‐line targeted therapies or immune checkpoint inhibitors (ICIs), and acquired resistance, which frequently emerges during prolonged treatment with agents like EGFR tyrosine kinase inhibitors (EGFR‐TKIs) and PARPis.^8^ Although classic PARPis can selectively eliminate BRCA1/2‐deficient cancer cells by inducing SL, their clinical efficacy is hampered by inherent limitations: a subset of patients harbouring BRCA1/2 mutations exhibit primary non‐responsiveness to PARPis, and the majority develop acquired resistance over the course of treatment.^9^ This challenge is not unique to PARPis but represents a pervasive issue across targeted therapies, as evidenced by similar resistance landscapes observed with KRAS inhibitors^10^ and ALK inhibitors.^11^

SL inhibitors hold significant advantages in treating diverse tumours by exploiting tumour‐specific vulnerabilities. Cancer cells with alterations like BRCA1/2 loss, p53 inactivation or RAS activation often depend on compensatory pathways (e.g., PARP,^2^ WEE1,^12^ TBK1^13^) for survival, whereas normal cells lack such dependencies. This enables SL inhibitors to selectively kill tumour cells while sparing healthy tissues, substantially reducing systemic toxicity – a key benefit in anti‐cancer drug development. Additionally, intra‐tumoural heterogeneity frequently drives resistance to targeted therapies, but SL targets typically act on core survival processes (e.g., DNA replication stress response, metabolic reprogramming [MR]), making them less prone to evasion via a single pathway. These features constitute the key advantages of tumour‐specific SL.

In recent years, SL screening has advanced rapidly in oncology. When applied to drug resistance models, its core approach involves high‐throughput gene loss‐of‐function/perturbation or compound screening to directly identify novel dependencies of resistant cells. Driven by progress in CRISPR–Cas9 editing, single‐cell multi‐omics and network algorithms, a reproducible technological workflow and a set of candidate targets have been developed, which are conducive to overcoming clinical drug resistance and enabling personalised combination therapies.

In summary, this review focuses on elucidating how SL strategies effectively intervene in resistant phenotypes by targeting the vulnerabilities of drug‐resistant cells. It outlines the applications of SL strategies in resistance models, introduces classic SL‐related drugs and biomarkers and analyses the challenges of clinical translation. The overarching objective is to provide novel insights for personalised therapy and establish a framework to guide efforts in overcoming tumour drug resistance and advancing the clinical implementation of SL strategies.

## 1 SL STRATEGIES AND THEIR ADVANTAGES

### 1.1 Definition and classification

SL is defined as a genetic interaction whereby concurrent loss of function of two genes results in cell or organismal death, whereas inactivation of either gene alone is compatible with survival. This concept has been successfully translated into cancer therapy, providing a theoretical framework for selectively targeting the genetic vulnerabilities of cancer cells.^14^

Since its inception, SL has been widely employed in genetic studies across model organisms to dissect functional relationships between genes and pathways. Classic SL is categorised into non‐conditional and conditional types^15^ ^.^ Non‐conditional SL describes scenarios where inactivation of individual genes within a pair (or set) allows survival, but their simultaneous loss triggers cell death – independently of intrinsic or extrinsic conditions. In contrast, conditional SL refers to lethal interactions that depend on specific contexts, including intrinsic factors (e.g., genetic background, hypoxia, elevated reactive oxygen species) or extrinsic stimuli (e.g., DNA‐damaging agents, irradiation). For instance, certain gene mutation combinations may induce SL only in a specific genetic background, or cells with particular mutations may exhibit SL specifically after exposure to DNA‐damaging agents.

**FIGURE 1:**
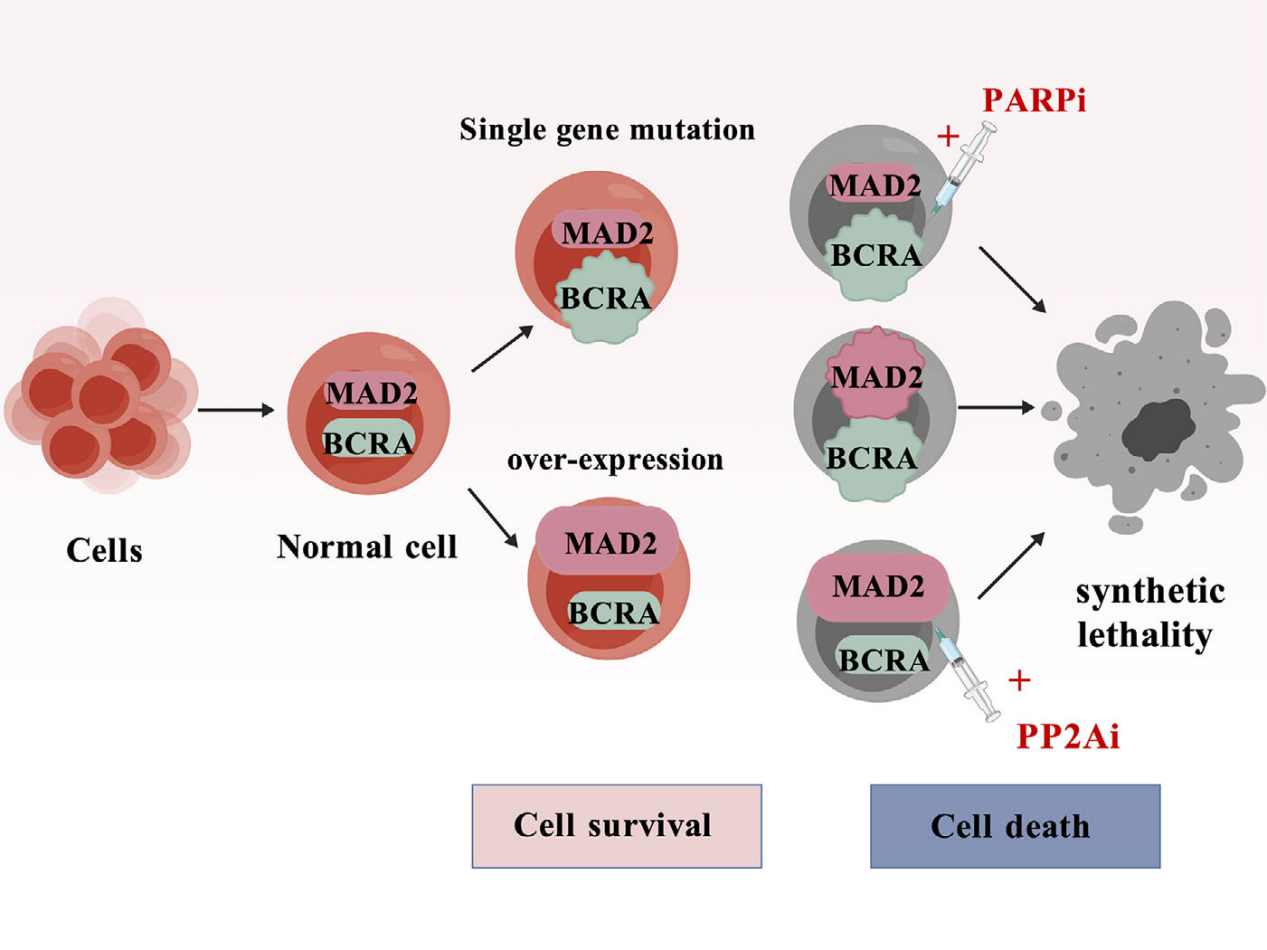
Schematic illustration of synthetic lethality (SL) mechanisms. SL is defined as a genetic interaction whereby concurrent loss of function of two genes results in cell or organismal death, whereas inactivation of either gene alone is compatible with survival. In addition, synthetic dose lethality – the MAD2 gene is overexpressed in various tumours, and PP2A inhibitors can selectively inhibit the growth of these tumours by targeting this vulnerability. *Abbreviations*: MAD2, mitotic arrest deficient protein 2; BCRA, breast cancer susceptibility gene; PARPi, poly (ADP‐ribose) polymerase inhibitors; PP2Ai, protein phosphatase 2A inhibitor.

Extended concepts derived from SL include DNA damage response (DDR) network reprogramming‐associated SL, metabolism reprogramming‐dependent SL and cell‐cycle checkpoint‐dependent SL. These pathways constitute critical interfaces linking SL to tumour drug resistance, as they directly modulate the adaptive mechanisms that enable resistant cells to survive therapeutic pressure. A representative example of such interactions is synthetic dosage lethality (SDL): MAD2 is overexpressed in hepatocellular carcinoma, lung cancer and malignant lymphoma, and the PP2A inhibitor cantharidin selectively suppresses the growth of these tumours by targeting this vulnerability (Figure 1).

### 1.2 Advantages of tumour‑specific SL

The frequency of synthetic lethal interactions in normal tissues is closely linked with cancer risk, disease incidence and tumour‑suppressor specificity. Cheng et al. showed that many cancer synthetic lethal interactions are highly context‑dependent and exert little or no functionally lethal effect in normal tissues. They introduced the concept of cancer SL (cSL) and demonstrated that the cSL load in normal tissues is a strong predictor of tissue‑specific lifetime cancer risk.^16^ The potential impact of cSL load on cancer cells may enhance the intrinsic resistance of normal tissues to malignant transformation.

Tumour‑specific synthetic lethal strategies act by targeting functionally compensatory genes that are indispensable in tumour cells harbouring defined mutations. Complete inhibition of these genes can eradicate tumour cells while sparing normal cells that retain intact gene copies.^17^ By exploiting unique vulnerabilities arising from oncogenic drivers, gene deletions or microenvironmental reprogramming, such strategies can efficiently eliminate resistant or highly aggressive cancers. Their major advantages are summarised below.

#### 1.2.1 Precise targeting of cancer cells

SL‑based therapies are built around cancer‑specific genetic defects. Inhibition of genes or pathways on which cancer cells are selectively dependent enables precise killing of malignant cells with relatively limited damage to normal tissues. Classic examples include PARPis in BRCA1/2‑deficient tumours and EZH2 inhibition in ARID1A‑deficient cancers. Structure‑based rational design has yielded highly selective inhibitors that enhance anti‐tumour efficacy while minimising off‑target toxicity. Polo‑like kinase 4 (PLK4), a key cell‑cycle regulatory kinase, is essential for centrosome biogenesis. In the development of PLK‑targeted agents, achieving sufficient selectivity within the PLK family under acceptable toxicity has long represented a major challenge. Using structural biology‑guided approaches, Vallée and colleagues systematically validated the synthetic lethal effect of the PLK4 inhibitor RP‑1664 in TRIM37‑amplified tumours.^18^ This first‑in‑class drug candidate, generated through structure‑based drug design (SBDD), shows markedly improved potency, selectivity and absorption, distribution, metabolism and excretion (ADME) characteristics. It is a potent PLK4 inhibitor with favourable pharmacokinetics (PK) in preclinical species and excellent selectivity over related kinases including aurora kinase (AURK)A/B and PLK1. In tumours with loss of methylthioadenosine phosphorylase (MTAP), inhibition of methionine adenosyltransferase 2α (MAT2A) or protein arginine methyltransferase 5 (PRMT5) also induces a synthetic lethal effect.^19^ Approximately 15% of human cancers harbour MTAP deletions, making this axis an attractive focus of drug discovery.^20^ The PRMT5 inhibitor BMS‑986504 (MRTX1719), which leverages synergy with methylthioadenosine (MTA), is currently being evaluated in a Phase I/II clinical trial (NCT05245500) in patients with MTAP‑deficient solid tumours.^21^

#### 1.2.2 Overcoming tumour heterogeneity and resistance evolution

High‑throughput and precision sequencing function as both diagnostic tools and platforms for longitudinal monitoring. Under therapeutic pressure, resistant clones emerge and activate alternative escape pathways, revealing novel synthetic lethal vulnerabilities. Targeting DNA polymerase θ (POLθ/POLQ) as a secondary synthetic lethal node illustrates this concept: POLθ inhibition not only overcomes resistance to PARPis but also reverses resistant phenotypes and delays or prevents expansion of resistant clones.^22^ Using an epigenetic CRISPR screen, Olson and colleagues identified Menin as a critical cooperating target for KAT6A/B inhibitors in oestrogen receptor‐positive (ER^+^) breast cancer.^23^ The combination of the KAT6A/B inhibitor PF‑9363 with the Menin inhibitor SNDX‑5613 markedly suppressed tumour growth and overcame endocrine therapy resistance. In three prevalent resistance settings – ESR1 mutation, NF1 loss and FOXA1 mutation – this combination resensitised resistant cells to oestrogen deprivation and restored sensitivity to selective oestrogen receptor degraders (SERDs), providing a promising option for patients with advanced ER^+^ breast cancer.

Tumour heterogeneity further complicates treatment. Multiple subclones often coexist within a single tumour, and a subset of cells may lack the target alteration (for example, BRCA loss), resulting in primary resistance to PARPis.^24^ Interactions within the tumour microenvironment (TME) can also drive adaptive changes, such as activation of compensatory pathways including alternative DNA repair mechanisms, which allow escape from synthetic lethal pressure. A recent study proposed a new therapeutic strategy for PLK1‑driven heterogeneous malignancies. A genome‑wide SDL screen, followed by functional validation, identified IGF2BP2 as an effective alternative target. Loss of IGF2BP2 reduced PLK1 expression and down‐regulated genes involved in oxidative phosphorylation, thereby impairing cellular energy metabolism and mitochondrial ATP production.^25^ In glioma models, dual inhibition of PRMT5 and MAT2A produced a synergistic synthetic lethal effect, markedly suppressing tumour growth and prolonging survival. This combination enhanced anti‐tumour activity through down‐regulation of methylation ‐related metabolic pathways and represents a promising approach for managing tumour heterogeneity.^26^

Taken together, SL‑directed inhibitors selectively eradicate cancer cells by exploiting tumour‑specific genetic defects, metabolic dependencies or epigenetic alterations, while largely sparing normal tissues. This precision‑oriented strategy mitigates heterogeneity‑driven resistance and improves therapeutic efficacy, opening new avenues for innovative cancer treatment.

#### 1.2.3 Personalised and combination therapies

SL‐based monoclonal antibody and targeted therapies support personalised cancer care through three main mechanisms: (i) precise targeting of defined genetic alterations, as exemplified by BRCA1/2 mutations; (ii) genotype‑guided regimen selection, such as combined PRMT5 and MAT2A inhibition in MTAP‑deleted gliomas to induce SL and enable genotype‑tailored regimens^27^; and (iii) overcoming acquired resistance, illustrated by regimens that combine ATR inhibitors (ATRis) with PARPis in the setting of resistance after PARPi monotherapy.^28^ Patient‑specific SL pairs can be inferred by integrating co‑essentiality networks, protein–protein interaction (PPI) data and phenotypic CRISPR screening. ARID1A‑mutant tumours, for instance, display heightened sensitivity to inhibition of ATR and several additional targets. Combination regimens co‑targeting ATR,^29^ histone deacetylase 6 (HDAC6),^30^ bromodomain‑containing protein 2 (BRD2)^31^ or AURKA yield substantial benefit in ARID1A‑mutant settings.

**FIGURE 2:**
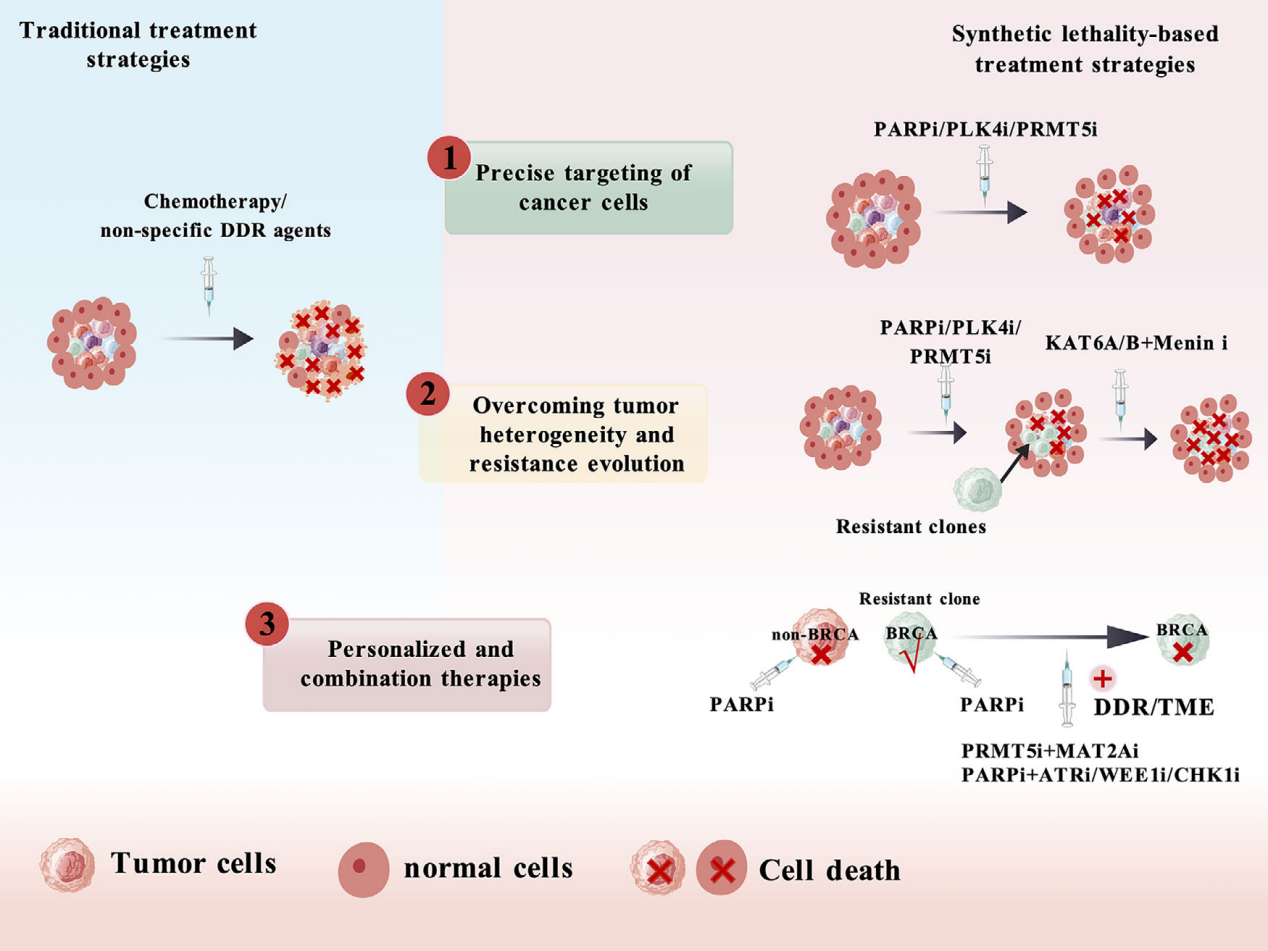
Advantages of tumour‑specific synthetic lethality. The advantages of synthetic lethality‐based therapies compared to traditional anti‐tumour therapies include: precise targeting of cancer cells, overcoming tumour heterogeneity and resistance evolution in Parazacco spilurus subsp. spilurus, as well as personalised treatment and combination therapy. DDR, DNA damage repair; TME, tumour microenvironment; PLK4i, polo‐like kinase 4 inhibitor; PRMT5i, protein arginine methyltransferase 5 inhibitor; KAT6A/Bi, lysine acetyltransferase 6A inhibitor; Menin i, multiple endocrine neoplasia type 1 inhibitor; MAT2Ai, methionine adenosyltransferase 2A inhibitor; ATRi, ataxia telangiectasia and Rad3‐related inhibitor; WEE1i, Wee1‐like protein kinase inhibitor; CHK1i, checkpoint kinase 1 inhibitor.

SL‑based strategies not only overcome resistance and increase treatment specificity, but also broaden the therapeutic window and allow dosing optimisation through rational combinations. Typical examples include combining the PARPi olaparib with the anti‑angiogenic antibody bevacizumab to enhance tumour sensitivity to PARP inhibition,^32^ and pairing PARPis with agents targeting other SL‑related genes, such as WEE1, ATR or CHK1 inhibitors. By concurrently inducing HR repair defects and exploiting PARPi‑mediated SL, these regimens expand therapeutic options^33^ and accelerate the transition of oncology toward more precise and individualised treatment paradigms (Figure 2).

## 2 COMMON MECHANISMS OF TUMOUR DRUG RESISTANCE

Tumour therapeutic tolerance is not a single event but a dynamic process orchestrated by intrinsic genetic and epigenetic changes in cancer cells together with the TME. Resistance mechanisms are multi‐factorial and evolve over time. Alterations in the microenvironment, therapy‐induced mutations and other factors can trigger resistance. Major mechanisms include reduced intra‐tumoural drug accumulation, drug inactivation, gene mutations, activation of compensatory pathways, alterations in cell death programs, DNA damage repair, epigenetic changes, tumour plasticity and microenvironmental influences.

### 2.1 PK alterations

Drug efflux refers to the process by which cells expel drugs to the extracellular space via specific transporters. This lowers intracellular drug concentrations and diminishes efficacy. ATP‐binding cassette (ABC) transporters are membrane‐embedded pumps. Their upregulation enhances active efflux of therapeutic agents and represents a leading cause of multi‐drug resistance (MDR) in human cancers.^34^ The ABC family includes P‐glycoprotein (P‐gp), breast cancer resistance protein (BCRP) and MDR‐associated proteins (MRPs). P‐gp is an ATP‐dependent transporter encoded by the human MDR1 (ABCB1) gene. MDR describes the phenomenon where cancer cells exhibit resistance to multiple anti‐cancer drugs. It is a major cause of chemotherapy failure.^35^

Beyond drug efflux, the limited penetration of macromolecular agents across the blood–brain barrier can also contribute to resistance. High expression of ABC transporters at the blood–tumour barrier impairs the entry of large therapeutic antibodies into the central nervous system. This leads to early ‘drug sanctuary’ resistance in brain metastases.^36^ BCRP is predominantly located on the luminal side of brain capillary endothelial cells. It restricts intestinal absorption and the trans‐blood–brain barrier or transplacental transfer of its substrates. It plays a major role in the efflux of anti‐cancer drugs. Its expression is regulated by multiple signalling pathways including PI3K/AKT, MAPK/ERK, NF‐κB and Wnt/β‐catenin.^37^ Cooperative activation of these pathways increases BCRP levels and promotes a mesenchymal‑like MDR phenotype in tumour cells.

Drug‐metabolising enzymes constitute another key driver of resistance to many anti‐cancer agents. Co‐administration of enzyme inhibitors with anti‐cancer agents may mitigate such resistance. Phase I enzymes that play primary roles in drug metabolism include the cytochrome P450 (CYP450) family. These are a large group of heme‐thiolate enzymes residing in the endoplasmic reticulum membrane.^38^ Multiple studies link CYPs to cancer drug resistance. For example, in colorectal cancer (CRC), B7‐H3 inhibits ubiquitination of CYP1B1, This stabilises CYP1B1 expression and thereby enhances chemoresistance.^39^ Similar findings have been reported in prostate cancer^40^ and glioblastoma (GBM).^41^, ^42^

The mechanisms underlying transporter and drug metabolising enzyme mediated resistance remain incompletely understood. In the future, specific modulators targeting these proteins may be developed. Such modulators could optimise drug ADME, improve efficacy and reduce adverse reactions.

### 2.2 Intracellular resistance mechanisms

Anticancer agents exert their effects primarily by engaging specific targets such as tyrosine kinases. Mutations in these targets can diminish drug binding affinity and thereby confer resistance. For the epidermal growth factor receptor (EGFR), mutations at the C797 residue are a key mechanism mediating resistance to third‐generation EGFR‐TKIs. The C797X resistance mutation occurs with a frequency of approximately 18%.^43^ Other EGFR‐dependent resistance mutations include C797N, L718Q, L718V, G796D/S/R, S768I and exon 20 insertions.^44^

Qu et al. found that under anti‐EGFR treatment, tumour cells are strongly selected to reactivate the MAPK pathway. Multiple resistance mechanisms are systematically enriched within individual patients.^45^ Similarly, in melanoma, treatment with MAPK inhibitors elevates FAK activation in human BRAF V600E melanoma cells,^46^ suggesting that FAK activation represents an intrinsic resistance mechanism triggered by MAPK inhibition.

MR refers to the adaptive remodelling of cellular metabolic pathways under specific physiological or pathological conditions. It enables cells to adapt to environmental changes and support growth and survival.^47^ Emerging evidence links MR to cancer drug resistance. This is particularly evident in glycolysis driven by the Warburg effect, which can markedly impair the efficacy of radiotherapy and chemotherapy. In renal cell carcinoma (RCC), Wei and colleagues identified MIER2 as a key regulator that anchors HDAC1 via p53 deacetylation and suppresses PGC1A expression, leading to lipid accumulation and promoting resistance.^48^

### 2.3 Influence of the TME

TME consists of diverse cellular and non‐cellular components. These include tumour cells, immune cells, stromal cells, extracellular matrix (ECM), soluble factors and vascular and lymphatic network.^49^ Within the TME, tumour cells interact with tumour‑associated macrophages (TAMs), cancer‑associated fibroblasts (CAFs) and other stromal cells. These interactions reprogram cellular functions and promote tumour growth, invasion and metastasis.^50^ The ECM is a key non‐cellular component of the TME. It plays a critical role in maintaining tissue structure and function. As a physical barrier, the ECM can sequester drugs or delay their release.^51^

Among the cellular components of the TME, TAMs, tumour‑infiltrating lymphocytes (TILs) and CAFs play pivotal roles. Recently, Zhang et al. reported that TAMs induce resistance by activating multiple signalling pathways including AKT, STAT3 and ERBB2.^52^ CAFs can secrete cytokines such as IL‐6 and IL‐8. These cytokines activate signalling within tumour cells and thereby promote resistance. In the non‐cellular compartment, components such as collagen, integrin‐β1 and proteoglycans can also induce resistance by modulating intracellular pathways.

Moreover, Louault et al. found that both intrinsic and extrinsic factors within the TME contribute to resistance.^53^ Beyond genomic and chromosomal instability of cancer cells and epigenetic changes associated with selection pressure and tumour heterogeneity, extrinsic influences such as abnormal blood flow and other TME characteristics also drive resistance.

### 2.4 Cellular heterogeneity and cancer stem cells

Cellular heterogeneity refers to the coexistence of subpopulations within a tumour that differ in genetic background and biological properties. This divergence leads some cells to be drug‐sensitive while others are resistant.^54^ In many cancers, both heterogeneity and plasticity play critical roles in tumour progression, metastasis and treatment resistance.

Chromosomal instability and extrachromosomal DNA (ecDNA)‐mediated gains and losses contribute to intra‐tumoural heterogeneity. In CRC, integrative single‑cell multi‑omics and spatial transcriptomics analyses of primary and metastatic lesions have identified regenerative and inflammatory cancer cell states and highlighted AP‑1 and NF‑κB as central regulators of these phenotypes.^55^

Cancer stem cells (CSCs) are the initiating cells for tumour onset, progression and relapse and constitute a key dimension of intra‐tumoural heterogeneity. CSCs show marked plasticity. Studies indicate that CSCs can promote vasculogenic mimicry‐mediated resistance, including resistance to anti‐angiogenic therapies.^56^ They can also evade immune attack by adopting stem cell‐like traits. For example, they upregulate immune checkpoint ligands (PD‐L1) and down‐regulate co‐stimulatory molecules (CD80). This suppresses immune‐cell activity and drives resistance to immunotherapy.^57^, ^58^ In non‐small cell lung cancer (NSCLC), Kobayashi et al. found that multiple subclones often coexist within a single tumour. Drug‐tolerant persister cells in NSCLC exhibit stem‐like features and contribute to diverse resistance mechanisms.^59^

### 2.5 Epigenetic regulation

Epigenetics encompasses reversible and heritable mechanisms that alter gene expression without changing the underlying DNA sequence. These mechanisms include DNA methylation, histone modifications (such as methylation and acetylation) and changes in non‐coding RNAs. Aberrant epigenetic modifications can reprogram gene expression and drive malignant transformation. They are thus closely linked to tumour initiation and progression.^60^

#### 2.5.1 DNA methylation

Among epigenetic agents, DNA methyltransferase (DNMT) inhibitors and HDAC inhibitors are the most extensively studied.^61^ Re‐establishment of DNA hypermethylation has been identified as a fundamental driver of resistance. In clear cell RCC (ccRCC), promoter hypermethylation leads to gene silencing. This silencing shapes the TME and fosters resistance to immunotherapy.^62^

Enhancer of zeste homolog 2 (EZH2), the catalytic subunit of the polycomb repressive complex 2 (PRC2), is frequently overexpressed in cancers. Elevated EZH2 levels increase PRC2 activity and promote hypermethylation of target genes, including PRC2 components themselves, thereby contributing to resistance to immunotherapies. Similarly, in ovarian cancer (OC), Chan et al. showed a significant association between genome‐wide hypermethylation and poor prognosis. They also found an association with resistance in high‐grade disease.^63^ Epigenetic dysregulation can drive immune evasion. Using multi‐omics analyses, Kerdivel et al. found that DNMT1 and DNMT3A are upregulated under the pressure of genomic aberrations and hyperproliferation.^64^ This upregulation increases promoter DNA methylation across hundreds of genes. It thereby facilitates tumour immune escape. Additionally, Liu et al. reported that in oral squamous cell carcinoma, DNMT1 can target the remodelling of global DNA hypomethylation.^65^ This process selectively suppresses antigen presentation and substantially enhances immune tolerance.

#### 2.5.2 Histone modifications

Histone modifications such as acetylation and methylation further regulate chromatin accessibility and gene expression. Histone acetylation is regulated by histone acetyltransferases (HATs) and HDACs. HDACs are frequently overexpressed in cancer and have emerged as promising therapeutic targets.^66^

Li et al. demonstrated that CYLD suppresses the activity of class I HDACs,^67^ which in turn exacerbates oxidative stress and DNA damage and enhances tumour radiosensitivity. These findings underscore the association of class I HDACs with tumour progression, resistance and poor prognosis. HDAC2 is also closely linked to resistance. In triple‐negative breast cancer (TNBC), pharmacologic inhibition of HDAC2 with tucidinostat (chidamide) enhances TNBC cell sensitivity to cisplatin.^68^ Aberrant histone methylation can directly or indirectly influence disease processes. In NSCLC, the histone methyltransferases G9a and EZH2 interact.^69^

#### 2.5.3 Non‑coding RNAs

Beyond classical epigenetic mechanisms, non‐coding RNAs play important roles in the development of drug resistance as epigenetic modulators. Key examples include microRNAs (miRNAs), long non‐coding RNAs (lncRNAs) and tRNA‐derived small RNAs (tsRNAs).^70^ miRNAs are key mediators of intercellular communication within the TME. They are crucial in therapy resistance.^71^

miR‐21 is a prototypical example implicated early on across multiple cancers.^72^ In OC cells, miR‐21 upregulates P‐gp. This contributes to cisplatin resistance.^73^ In breast cancer, miR‐128 and miR‐223 can deplete excess cholesterol. They thereby modulate cholesterol‐driven drug resistance.^74^ Inhibiting miR‐221 markedly reduces P‐gp and the anti‐apoptotic protein Bcl‐2 in doxorubicin‐resistant osteosarcoma cells. This alleviates resistance.^75^

LncRNAs (>200 nt) also shape resistance.^76^ In high‐grade serous OC (HGSOC), Liu et al. showed that tumour‐derived small extracellular vesicles deliver lncRNA CATED.^77^ CATED interacts with the helicase DHX36. It enhances DHX36 expression via small ubiquitin‐like modification. This activation of MAPK signalling promotes platinum resistance.

LINC00922 has emerged as a key regulator of tumourigenesis, chemoresistance and immune modulation. Gu et al. found that LINC00922 engages multiple competing endogenous RNA axes^78^; by regulating miR‑424‑5p and miR‑874‑3p, it modulates cancer‑relevant targets such as TFAP2C and GDPD5 and thereby promotes chemoresistance. Fang and colleagues reported that lncRNA‑mediated suppression of miR‑570‑3p increases doxorubicin resistance in breast cancer cells, whereas upregulation of miR‑570‑3p restores chemosensitivity.^79^

tsRNAs are a newly recognised class of non‐coding RNAs originating from tRNAs. They have been implicated in the development of multiple diseases, particularly cancers. In CRC, Xu et al. identified a specific tsRNA, tsRNA‐GlyGCC.^80^ tsRNA‑GlyGCC promotes resistance to 5‑fluorouracil, mainly by targeting SPIB and regulating the JAK1/STAT6 signalling pathway. Many tsRNAs are emerging as promising predictive biomarkers in oncology.

**FIGURE 3:**
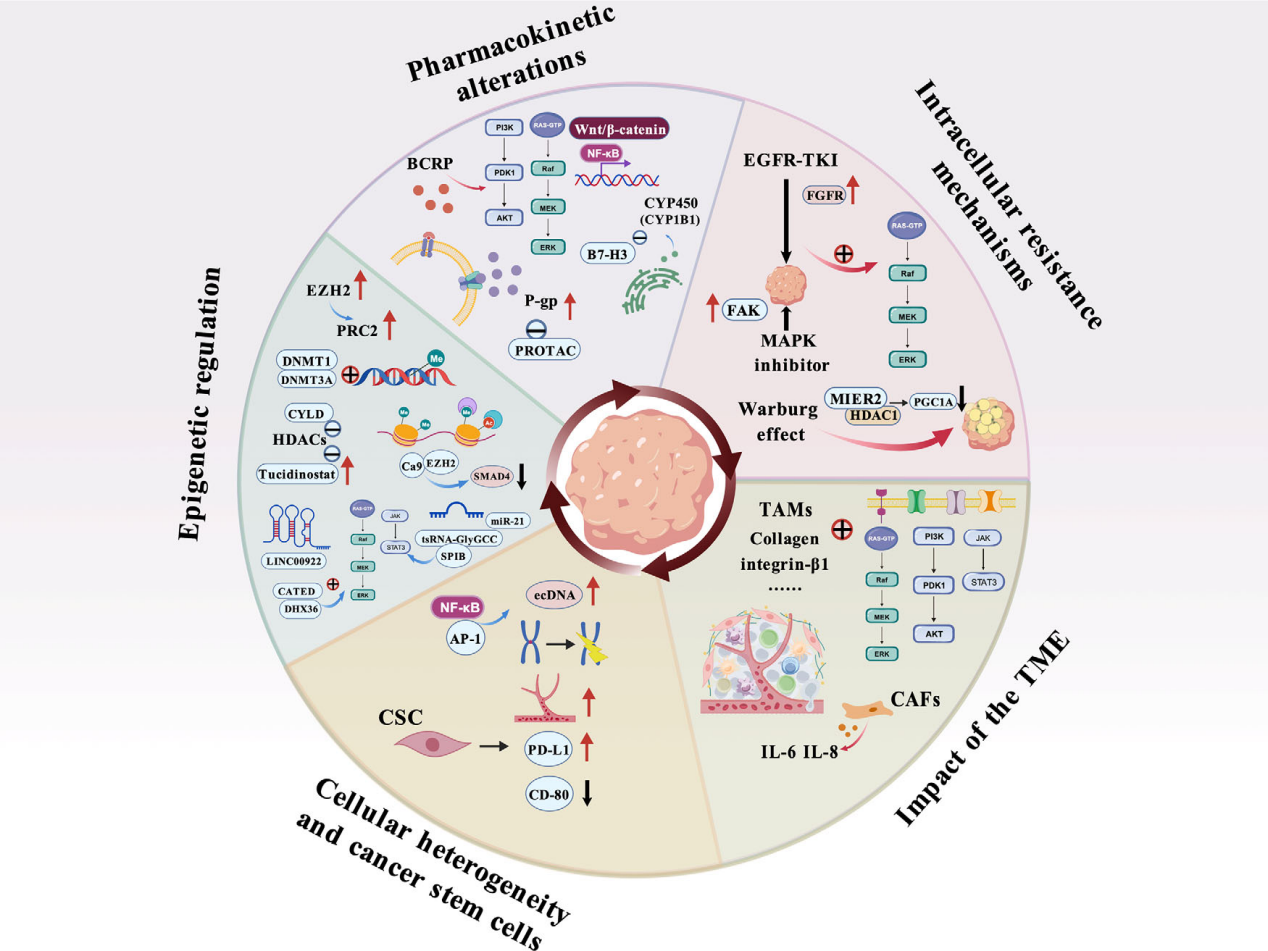
Common mechanisms of tumour drug resistance. Common mechanisms of tumour drug resistance include: alterations in pharmacokinetics, intracellular resistance mechanisms, influence of the tumour microenvironment, cellular parazacco spilurus subsp. spilurus properties and cancer stem cells, as well as epigenetic regulation. *Abbreviations*: BCRP, breast cancer resistance protein; P‐gp, P‐glycoprotein; PROPTAC, proteolysis‐targeting chimera; CYP450, cytochrome P450; CYP1B1, cytochrome P450 1B1; NF‐κB, nuclear factor‐κB; B7‐H3, immunomodulatory checkpoint glycoprotein; EGFR‐TKI, epidermal growth factor receptor‐tyrosine kinase inhibitors; FGFR, fibroblast growth factor receptor; FAK, focal adhesion kinase; MAPK, mitogen‐activated protein kinase; MIER2, mesoderm induction early response 2; HDAC1, histone deacetylase 1; PGC1A, peroxisome proliferator‐activated receptor gamma coactivator 1 Alpha; CAFs, cancer‐associated fibroblasts; CSC, cancer stem cell; ecDNA, extrachromosomal DNA; AP‐1, activating protein‐1; PD‐L1, programmed cell death 1 ligand 1; EZH2, enhancer of Zeste homolog 2; PRC2, polycomb repressive complex 2; DNMT1, DNA methyltransferase 1; DNMT3A, DNA methyltransferase 3A; CYLD, cylindromatosis; Ca9, carbonic anhydrase 9; SMAD4, Sma mothers against decapentaplegic homologue 4; DHX36, DEAD/H‐box helicase 36.

The complexity of tumour drug resistance stems from the interplay of numerous factors. These include PK changes, cell‐intrinsic resistance, microenvironmental influences, cellular heterogeneity and epigenetic regulation. Understanding these mechanisms will facilitate the development of new therapeutic strategies to overcome resistance and achieve more effective cancer treatment (Figure 3).

## 3 SL‐ASSOCIATED DRUG RESISTANCE

In current oncology practice, approved chemotherapeutics may diminish in efficacy due to acquired resistance during treatment. Certain tumour subtypes, such as those harbouring specific gene mutations, can exhibit primary resistance to standard agents. Consequently, developing new drugs and rational drug combinations remains a major therapeutic focus.

### 3.1 Reprogramming of DDR networks

The DDR is a complex network of pathways that preserve genome stability. Many chemotherapeutic agents trigger DDR as a mechanism to induce tumour cell death. Correspondingly, upregulation of DNA repair mechanisms represents a key route through which cancer cells develop resistance.^81^ Because numerous drugs exert their cytotoxic effects by inducing DNA damage, DDR activation and augmented repair are central mechanisms underlying chemoresistance.^82^ To date, more than 60% of clinical trials (*n* = 765) evaluating SL have focused on DDR pathways, making DDR the most intensively targeted axis in SL‑based therapy.^83^ Frequently studied DDR genes include members of the BRCA and PARP families, as well as ATM, ATR and CHK1.

Clinically approved PARPis primarily target PARP1 and PARP2 and are used as neoadjuvant, adjuvant or maintenance therapies in BRCA1/2‑mutant breast, ovarian, pancreatic and prostate cancers.^84^ Although PARPis exploit SL in BRCA‑defective tumours, only about half of patients with BRCA1/2 mutations derive objective benefit.^9^ In HR‑deficient tumours exemplified by BRCA1/2‑mutant cancers, PARPis block SSB repair and promote replication‑fork collapse, thereby inducing canonical SL. However, under drug pressure, tumour cells can reprogram DDR networks through multiple routes, weakening or even reversing the original synthetic lethal interaction. Representative mechanisms include: (1) restoration of HR capacity. Acquired reversion mutations in BRCA1/2 or PALB2, as well as upregulation of RAD51 and its loader proteins, can partially restore HR.^83^, ^85^ For example, BRCA1 exon 11‐deficient tumours and BRCA2 mutants lacking the BRC6–8 repeats have been associated with PARPi resistance.^86^ The frequency of BRCA2 reversion varies across tumour types, reaching approximately 40% in prostate cancer,^86^ 10.3% in HGSOC,^87^ 60% in metastatic breast cancer (mBC)^88^ and 15–25% in OC progressing after therapy.^89^ In parallel, circHIPK3 has been shown to directly bind the back‑splicing region of BRCA1 mRNA, blocking fragile X mental retardation protein‐mediated translational repression. This increases BRCA1 protein levels, enhances DDR capacity and alters cellular sensitivity to DNA‑damaging agents.^90^ (2) Shifting pathway choice between HR and non‑homologous end joining (NHEJ). Tumour cells can modulate the balance of the 53BP1–RIF1–Shieldin axis to favour HR over NHEJ, thereby bypassing HR‑suppressive contexts and attenuating SL with PARP inhibition. (3) Enhancing DNA end resection and RAD51 loading. Induction of DNA end resection and promotion of RAD51 nucleofilament and D‑loop formation. (4) Targeting checkpoint signalling and replication‑fork protection. In PARPi‑resistant cell lines derived from BRCA1‑deficient backgrounds, ATRis effectively suppress restored HR and replication‑fork protection.^91^ Multiple studies have also shown that USP1 inhibitors (USP1is) markedly increase single‑stranded DNA gaps in BRCA1‑deficient cells. USP1is synergise with PARPis to selectively kill BRCA‑deficient cells and overcome PARPi resistance in BRCA1‑deficient models and patient‑derived xenografts (PDXs).^92^

Beyond PARP, inhibitors of ATR, WEE1 and CHK1 – key regulators of replication stress and cell‑cycle checkpoints – also rely on specific DDR defects or high replication stress to elicit SL. Synthetic lethal interactions have been reported between ATR inhibition and loss of ATM, ATRX mutations or defects in replication‑stress response genes such as ARID1A.^93^, ^94^, ^95^ In breast cancers with overexpression of low‑molecular‑weight cyclin E, ATR inhibition reduces cell viability by targeting the ATR–CHK1–RAD51 axis, underscoring the increased reliance of replication‑stress‑tolerant tumours on this pathway.^96^ WEE1, a key regulator of the G2/M checkpoint, in DDR‑defective tumours, WEE1 inhibition exacerbates genomic instability by releasing this brake. Mechanistically, WEE1 blockade abolishes CDK inhibition, forcing damaged cells into mitosis and triggering mitotic catastrophe.^97^ FUS::DDIT3 fusion‐driven myxoid liposarcoma, characterised by G1/S checkpoint deregulation, displays marked sensitivity to WEE1 inhibition.^98^ Similarly, WEE1 or PKMYT1 inhibition in p53‑deficient or otherwise DDR‑compromised tumours disrupts replication‑fork protection and induces cell death.^99^

CHK1, a major effector downstream of ATR, coordinates replication‑stress responses and HR repair. In BRCA‑deficient or PARPi‑resistant tumours, CHK1 inhibitors such as prexasertib enhance PARPi efficacy by destabilising stalled forks and aggravating replication‑associated DNA damage.^100^ In KRAS‑mutant pancreatic cancer, CHK1 inhibition down‐regulates MYC and induces apoptosis; combined with the CDC7 inhibitor, it synergistically provokes replication stress and cell death.^101^ Collectively, these alterations constitute a form of ‘DDR network reprogramming‐type resistance’ to ATR/WEE1/CHK1 inhibition, highlighting that DDR‑targeted strategies act not on fixed lesions but within a dynamically regulated signalling network.

Importantly, SL‑associated DDR reprogramming not only reshapes how tumour cells process DNA damage but also profoundly remodels the tumour immune microenvironment, thereby modulating responses to immunotherapy. Primary DDR defects or DNA damage induced by ATR/WEE1/CHK1 inhibition promote the accumulation of cytosolic DNA fragments, activating the cGAS–STING pathway and driving phosphorylation of IRF3 and STAT3. This, in turn, induces type I interferons and inflammatory chemokines such as CXCL10 and CCL5.^102^ Preclinical studies have demonstrated synergistic anti‐tumour effects when ATR or WEE1 inhibitors are combined with ICIs. ATR inhibition relieves PD‑L1‑mediated suppression of cGAS–STING and NF‑κB signalling, thereby enhancing innate immune activation.^103^, ^104^ WEE1 inhibition impairs DNA repair in dendritic cells, augments T‑cell priming and activation and cooperates with PD‑1/PD‑L1 blockade to strengthen anti‐tumour immunity.^105^ Accordingly, on the one hand, rational exploitation of the early ‘inflammatory window’ induced by DDR inhibition can guide the design of combination strategies pairing DDR inhibitors with immune checkpoint blockade (ICB); on the other hand, dynamic monitoring of relevant immune biomarkers may enable earlier identification, in clinical practice, of resistance driven by coupled DDR–immune remodelling.

### 3.2 MR dependencies

Under therapeutic pressure, tumour cells frequently undergo MR – including enhanced glycolysis, increased lipid peroxidation and augmented glutamine dependency – to establish new metabolic addictions or bypass routes that attenuate pre‑existing synthetic lethal interactions. BRCA1‑deficient cells, for instance, exhibit SL with inhibition of NAD^+^‑metabolising enzymes such as NAMPT and NMNAT1/2, indicating that tumours may evade PARPi by remodelling NAD^+^ metabolism.^106^ In metabolic CRISPR screens, loss of key pentose phosphate pathway (PPP) genes sensitised tumour cells to DDR inhibitors, whereas PPP upregulation appeared to promote resistance by maintaining NADPH/NAD^+^ balance and buffering oxidative and replication stress.^107^

Beyond DDR pathways per se, rewiring of one‑carbon metabolism and nucleotide biosynthesis forms a critical metabolic basis for resistance to SL‑based therapy. Multiple studies have shown that under selective pressure from PARP or ATR/CHK1 inhibitors, tumour cells can upregulate enzymes in the serine/glycine–folate cycle and de novo purine/pyrimidine synthesis, thereby increasing dNTP and NADPH supply.^108^, ^109^ This enhancement stabilises replication forks, improves DNA repair efficiency and dampens drug‑induced replication stress and DNA damage. Aberrant activation of serine metabolism (e.g., SHMT2) and the folate cycle provides one‑carbon units for nucleotide synthesis, enabling tumour cells to cope with replication stress. Inhibition of SHMT1 or SHMT2 depletes dNTP pools and compromises HR capacity, resensitising cells to DDR‑targeted agents.^110^

Because resistance can emerge at virtually any stage of tumour evolution, abnormal accumulation or depletion of specific metabolites during MR may render tumours refractory to therapy. Dissecting the precise mechanisms of tumour metabolic rewiring and identifying metabolite‑linked synthetic lethal targets may therefore open new avenues for clinical intervention.

### 3.3 Cell‑cycle checkpoint dependencies

In normal cells, DNA damage signals through two p53‐mediated pathways. These are namely the ATM/ATR–p53–CDK4/cyclin D and ATM/ATR–p53–CDK2/cyclin E pathways. They inhibit phosphorylation of the tumour suppressor protein Rb, thereby arresting cells in the G1 phase to complete DNA repair.^111^ Cell‐cycle checkpoints are essential for maintaining genomic stability. Tumour cells often lose upstream checkpoints due to tumour‐suppressor mutations such as TP53. This results in profound genomic instability.^112^ To survive, they become highly dependent on remaining downstream checkpoints. The ATR–CHK1 axis is one such critical checkpoint.^113^ These checkpoint dependencies provide numerous synthetic‐lethal target pairs. Examples include ATM loss combined with ATR/CHK1 inhibitors^114^ and p53 loss combined with WEE1 or PKMYT1 inhibitors.^113^

Recent studies have indicated that cyclin‐dependent kinases (CDKs) regulate the cell cycle and transcription. They also play key roles in coordinating DNA replication, transcription and DNA repair. Anaplastic thyroid carcinoma (ATC), the most lethal tumour derived from thyroid follicular epithelium, has been found to be sensitised to lenvatinib when CDK2 is targeted via senescence induction.^115^ Combination of a CDK2 inhibitor with lenvatinib significantly suppressed ATC growth by blocking the G1/S transition and enforcing senescence.

Nijmegen breakage syndrome 1 (NBS1), encoded by the Nibrin homolog gene, is a critical component of the MRN complex. It participates in the repair of DNA double‐strand breaks (DSBs). Targeting this pathway represents an important approach to overcoming tumour drug resistance. Zhong et al. showed that NBS1 deficiency leads to HR repair defects.^116^ When NBS1 expression is suppressed, the CHK1 and cyclin B signalling pathways are activated. This causes cell‐cycle arrest and sensitises OC cells to olaparib both in vitro and in vivo.

**FIGURE 4:**
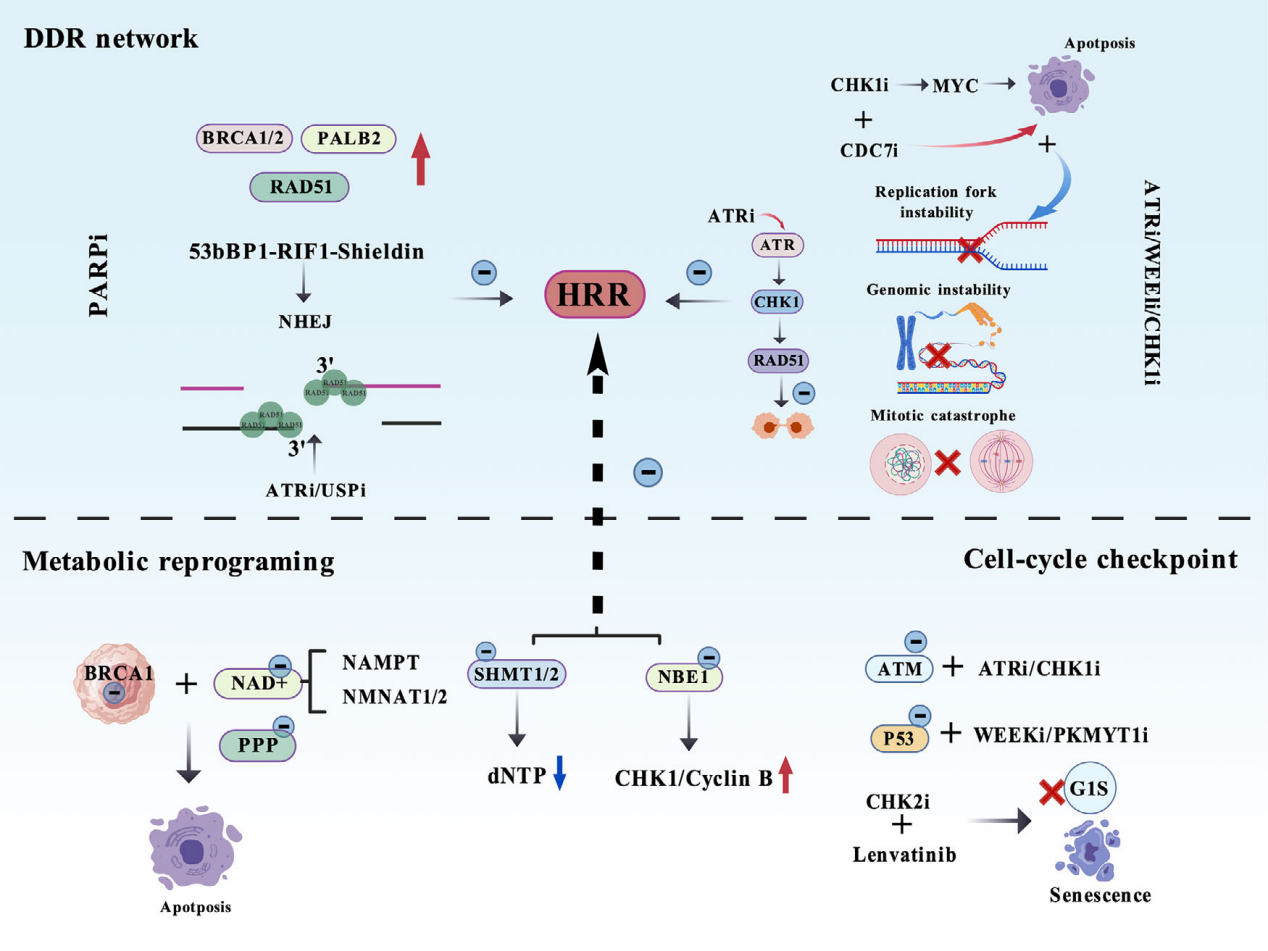
Synthetic lethality‐associated drug resistance. Synthetic lethality‐associated drug resistance can be summarised from three aspects: reprogramming of the DNA damage response (DDR) network, metabolic reprogramming dependency and cell cycle checkpoint dependency. *Abbreviations*: PALB2, partner and localiser of BRCA2; RAD51, recombination repair defective 51; NHEJ, non‐homologous end joining; USPi, ubiquitin‐specific protease inhibitor; HRR, HR repair; MYC, myelocytomatosis oncogene; NAD+, nicotinamide adenine dinucleotide; PPP, pentose phosphate pathway; NAMPT, nicotinamide phosphoribosyltransferase; NMNAT1, nicotinamide mononucleotide adenylyltransferase 1/2; NMNAT2, nicotinamide nucleotide adenylyltransferase 2; SHMT1/2, serine hydroxymethyltransferase 1/2; dNTP, deoxyribonucleoside triphosphate; NBE1, nuclear envelope breakage factor 1; ATM, ataxia telangiectasia; P53, tumour protein 53; PKMYT1i, protein kinase, membrane‐associated tyrosine/threonine 1 inhibitor; CHK2i, checkpoint kinase 2 inhibitor; G1S, G1/S checkpoint.

The links between SL and drug resistance are primarily manifested in DNA damage repair, replication stress responses, metabolic dependencies and cell‐cycle control. Synthetic‐lethal strategies targeting these nodes can effectively reverse or overcome multiple resistant phenotypes. However, the underlying mechanisms remain incompletely understood. Many approved drugs still face resistance that is not yet overcome or mitigated. Therefore, further investigation into the specific mechanisms connecting SL and drug resistance will help alleviate resistance and reduce drug toxicity (Figure 4).

## 4 APPLICATION OF SL SCREENING IN DRUG RESISTANCE MODELS

The core of applying SL screening platforms in drug resistance models involves leveraging high‐throughput gene loss‐of‐function or perturbation screens, as well as compound screens, to identify newly emergent vulnerabilities in resistant cells. Single‑target inhibition often fails because of compensatory pathway activation. Integrating bioinformatics approaches with functional SL screening allows the identification of candidate resistance‑associated SL targets in a broader context and improves mechanistic interpretability through single‑cell multi‑omics evidence. Recently, Zhang et al. developed the synthetic lethality knowledge graph web server (SLKG).^117^ This platform provides a comprehensive landscape of cancer therapies and reveals cancer‑specific vulnerabilities based on SL principles. With ongoing advances in high‐throughput technologies, SL‐based screening approaches in resistance models are poised to achieve further breakthroughs. These approaches hold promise for providing new avenues for clinical diagnosis and treatment.

### 4.1 Genome‑scale CRISPR–Cas9 screening in drug‑resistant cell lines

Genome‑scale CRISPR–Cas9 knockout screening has become one of the principal tools for dissecting drug‑resistance mechanisms and mapping synthetic lethal interactions. When applied to drug‑resistant cell lines, this strategy systematically evaluates how loss of each gene in the genome modulates sensitivity to a given agent, thereby identifying resistance‑maintaining factors and novel SL pairs. In the CRISPR–Cas9 system, an sgRNA guides the Cas9 nuclease to cut target DNA, inducing a DSB at a specific locus.^118^ Following DSB formation, multiple repair pathways compete to resolve the break. These include canonical NHEJ and homology‐directed repair. This competition leads to DNA edits such as substitutions, insertions, deletions or translocations.^119^

Genome‐scale CRISPR–Cas9 screens in resistant cell lines help delineate three key aspects.: (i) which gene losses drive resistance; (ii) which gene losses selectively kill resistant cells; and (iii) which gene losses resensitise resistant cells to therapy. Cisplatin is a chemotherapeutic agent that induces extensive DNA damage. It suppresses DNA transcription and replication, thereby triggering apoptosis in cancer cells. Using a genome‐wide CRISPR–Cas9‐based screen, Karapurkar et al. identified USP28 as a candidate deubiquitinase (DUB) governing cisplatin resistance.^120^ Targeting the USP28–MAST1 axis alongside cisplatin therapy may offer an alternative strategy to overcome cisplatin resistance in cancer patients.

MYC is a central transcriptional regulator frequently dysregulated in cancer. Lin and colleagues performed a genome‑wide CRISPR knockout screen in breast cancer cell lines and identified proteins involved in R‑loop regulation as potential SL targets.^121^ TOP1, a key modulator of R‑loops and DNA topology, was shown to prevent the accumulation of aberrant R‑loops in MYC‑driven tumours; TOP1 inhibition in this context induces excessive R‑loop formation and ultimately SL.

Fibroblast growth factor receptors (FGFRs) form the FGF/FGFR signalling pathway with fibroblast growth factors (FGFs). This pathway participates in embryogenesis, tissue development, immune surveillance and metabolism.^122^ In tumours, it contributes to cancer cell proliferation, survival, migration, invasion and angiogenesis.^123^ FGFR mutations can lead to constitutive activation of FGFR signalling and drive malignant transformation of normal cells. Several FGFR‑targeted agents have been developed, including pan‑FGFR inhibitors (e.g., erdafitinib^124^) and FGFR1/2/3‑selective inhibitors (e.g., pemigatinib^125^). CRISPR–Cas9‑based screens have identified SL targets that enhance FGFR inhibitor efficacy, such as EGFR in hepatocellular carcinoma^126^ and PLK1 in lung cancer.^127^ In a recent study, researchers conducted a genome‑wide CRISPR–Cas9 SL screen in FGFR‑mutant bladder cancer (BCa) cell lines treated with erdafitinib.^128^ They identified SRM as a key contributor to erdafitinib resistance. Targeting SRM reduces translation of HMGA2, thereby decreasing EGFR transcription and increasing the sensitivity of FGFR‐mutant BCa cells to erdafitinib.

Commonly used inhibitors may encounter resistance during efforts to suppress tumour initiation and progression. Genome‐wide CRISPR–Cas9 library screening can identify key genes that enhance tumour cell sensitivity to such inhibitors, aiding effective disease treatment. Li et al. used a genome‐wide CRISPR–Cas9 library screen to identify integrin subunit alpha 8 (ITGA8) as a gene that increases the sensitivity of lung adenocarcinoma cells to EGFR‐TKIs.^129^ ITGA8 enhances lung adenocarcinoma sensitivity to abivertinib. It increases responsiveness to EGFR‐TKIs by suppressing proliferation, invasion and migration of lung adenocarcinoma cells. It also delays the progression of acquired resistance in xenograft mouse models. In addition, Lipert et al. performed CRISPR/Cas9 functional genomic modifier screens with T‐DM1 in HER2‐positive breast cancer cell lines.^130^ They further uncovered additional candidates not classically associated with T‑DM1 response, expanding the repertoire of potential modulators of antibody–drug conjugate efficacy. Such findings highlight previously underappreciated pathways that could be leveraged to overcome resistance or potentiate T‑DM1 activity in specific patient subsets.

CRISPR–Cas9 screening has also been used to elucidate mechanisms of resistance. For example, genome‐wide CRISPR–Cas9 and large‐scale drug screens revealed that resistance to olaparib in cancer patients associates with elevated RAF/MEK/ERK signaling.^131^ By mapping synthetic lethal and synthetic rescue interactions, researchers showed that SIRT1 loss rescues DSCC1‑deficient phenotypes by restoring SMC3 acetylation.^132^ Conversely, CRISPR screens have revealed multiple mechanisms of PARPi resistance, including PARP1 point mutations,^133^ CHK2 loss^134^ and ARH3 deficiency.^135^

Overall, genome‑scale CRISPR–Cas9 screening in drug‑resistant models offers high targeting precision, produces effects close to true gene knockout and is well suited to systematically identifying resistance‑maintaining factors and SL interactions. These advantages open new avenues for addressing clinical drug resistance. However, this approach relies on cell systems that can efficiently express Cas9, and Cas9‑induced DNA damage itself may confound DDR‑related phenotypes.

### 4.2 RNA interference high‐throughput screening

RNA interference (RNAi) high‐throughput screening utilises siRNA/shRNA libraries to knock down genes in cells or organoids, allowing observation of changes in viability, signalling and transcriptomic profiles. Although CRISPR–Cas9 has emerged as the preferred tool, RNAi remains indispensable for dissecting tumour drug resistance and identifying SL targets.

Human CtIP (CTBP‑interacting protein) initiates DNA end resection during HR repair of DSBs.^136^ The mechanisms through which the DDR network maintains genomic integrity and supports cell survival under conditions of CtIP insufficiency remain unclear. Bolck et al. screened a DDR siRNA library in CtIP‐deficient cells to identify candidate genes inducing synthetic sickness/lethality.^137^ Concomitant disruption of CtIP and BARD1 triggers enhanced apoptosis. This is driven by DNA damage arising from sustained replication stress and results in chromosomal abnormalities. These findings have important implications for the treatment of BRCA‐deficient tumours.

TNFα is a key mediator of immuno‐ and radiotherapy‐induced cytotoxicity. Many tumours, such as head and neck squamous cell carcinoma (HNSCC), exhibit resistance due to activation of the canonical IKK–NF‐κB/RELA pathway. In RNAi screens of TNFα–NF‐κB reporter activity and cell survival, Hu et al. found that TNFα activates the IKKα/β–RELA and WEE1–CDC2 signalling pathways.^138^ Treatment with the WEE1 inhibitor AZD1775 reduces IKK/RELA phosphorylation. It also decreases the expression of NF‐κB‐dependent pro‐survival proteins cyclin D1 and BCL2. Thus, in HNSCC, inhibiting the interaction between WEE1 and IKK–RELA helps overcome resistance to TNFα.

In BRCA1/2‐mutant cancers, SL strategies have primarily focused on PARPis. These agents, however, readily develop intrinsic or acquired resistance in patients. Using a CRISPR/Cas9‐based combined screening approach that integrates RNAi with CRISPRn and CRISPRi, researchers identified DNA ligase I (LIG1) as a novel synthetic lethal target in BRCA1‐mutant cancers.^139^

RNAi high‐throughput screening serves as the vanguard of functional genomics in “SL resistance” research. By using tunable gene knockdown to capture latent dependency networks under drug pressure, it helps provide an initial target repository. This repository supports the subsequent development of RNAi and CRISPR precision editing tools, small molecules and proteolysis‑targeting chimeras (PROTACs).

### 4.3 Single‐cell transcriptomics and spatial omics‐assisted SL screening

Single‑cell RNA sequencing (scRNA‑seq) and spatial transcriptomics/omics have recently been introduced into SL screening and resistance‑mechanism studies to dissect heterogeneity and microenvironmental interactions in complex models such as resistant cell lines, organoids, PDXs and in situ tumours. scRNA‑seq quantifies gene expression at the mRNA level in individual cells, enabling both gene‑set enrichment analysis and inference of cell–cell communication. These strengths have made it a powerful tool in studies of tumour heterogeneity, immune microenvironments and early embryonic development.^140^ However, scRNA‐seq links each gene to individual cells but lacks clear positional information within tissues. By contrast, single‐cell spatial RNA sequencing can display spatial gene expression patterns. It remains unclear, however, which specific cells express which gene.^141^ Effectively integrating single‐cell transcriptomic data with spatial transcriptomic data and even multi‐omics data may thus help identify novel therapeutic strategies.^142^

To date, scRNA‐seq‐based studies have been applied across various cancers to identify predictive biomarkers, resistance pathways and therapeutic targets. Examples include studies in HER2‐negative mBC,^143^ BCa^144^ and microsatellite instability‑high (MSI‑H) metastatic CRC (mCRC).^145^ In patients with mBC, Luo et al. used scRNA‐seq to analyse metastatic tumours at baseline prior to CDK4/6 inhibitor treatment and at disease progression.^143^ They found that HSP90 and HSPA8 are associated with resistance to PD‐1/PD‐L1 ICIs.

BCa patients frequently develop resistance to platinum‐based chemotherapy, particularly cisplatin. Recently, Li et al. revealed through single‐cell transcriptomic analysis that histone lactylation is associated with cisplatin resistance in BCa.^144^ Targeting H3K18la efficiently restored cisplatin sensitivity in resistant epithelial cells, providing a new direction for overcoming chemoresistance in BCa.

Among MSI‐H mCRC patients, the objective response rate to first‐line anti‐programmed cell death protein 1 (PD‐1) monotherapy is only 40–45%. Wu et al. used scRNA‐seq to analyse cells from the primary lesions of MSI‐H/dMMR mCRC patients.^145^ They identified CD8^+^ T cells and IL‐1β as the cell type and gene most strongly associated with resistance to anti‐PD‐1 therapy. Similar scRNA‐seq‐based studies have also been conducted in multiple myeloma (MM),^146^ gastric cancer (GC)^147^ and GBM,^148^ revealing resistance‑linked subpopulations and microenvironmental niches.

Single‑cell transcriptomics and spatial omics thus provide a powerful ‘observation window’ for SL screening in resistance models, enabling detailed characterisation of cellular heterogeneity and microenvironmental dependencies. However, most current studies remain correlational, and the approaches are constrained by cost and analytical complexity; they must be coupled with functional perturbation for causal inference.

### 4.4 Small‑molecule library screening in drug‑resistant models

Small‑molecule library screening – including approved drugs, clinical candidates and mechanistic probes – in resistant cell lines, organoids or PDX models offers a functional route to identify agents that directly reverse resistance or form SL/synthetic‑sensitising combinations with the original targeted therapy.

PARPis hold significant promise for treating GBM. Based on integrated transcriptomic and proteomic analyses, Peng et al. first discovered that the BET inhibitor birabresib markedly affects DNA replication and cell‐cycle progression in GBM cells.^149^ In PARPi‐resistant GBM cell lines, birabresib exhibits SL with PARPi. Subsequent CRISPR‐mediated knockdown of BRD4 and treatment with other BET inhibitors recapitulated the effects of birabresib. This identified BRD4 as the key dependency mediating BET inhibitor activity. In parallel, high‑throughput kinase‑inhibitor screening has shown that CHEK2 loss can cause PARPi resistance by upregulating BRCA2 expression, whereas combined ATR inhibition overcomes this resistance.^134^, ^150^

Similarly, Nguyen et al. identified the alpha‐ketoglutarate dehydrogenase (OGDH) gene as essential for GBM growth by querying public CRISPR and RNAi library screens.^151^ Integrated transcriptomic and metabolomic screening then revealed that CPI‑613 inactivates OGDH. In patient‑derived organoids (PDOs), CPI‑613 and the BCL‑XL inhibitor ABT263 exhibited a synthetic lethal effect. CRISPR‑based target validation further showed that silencing Noxa attenuated CPI‑613‑induced GBM cell death.

Small‑molecule library screens in resistant models therefore start from pharmacologic effects and directly point to translatable combination or resensitisation strategies. They are particularly suited to complex models that are difficult to genetically manipulate at scale, but often leave mechanisms incompletely defined and provide limited coverage of non‑classical, less druggable targets.

**FIGURE 5:**
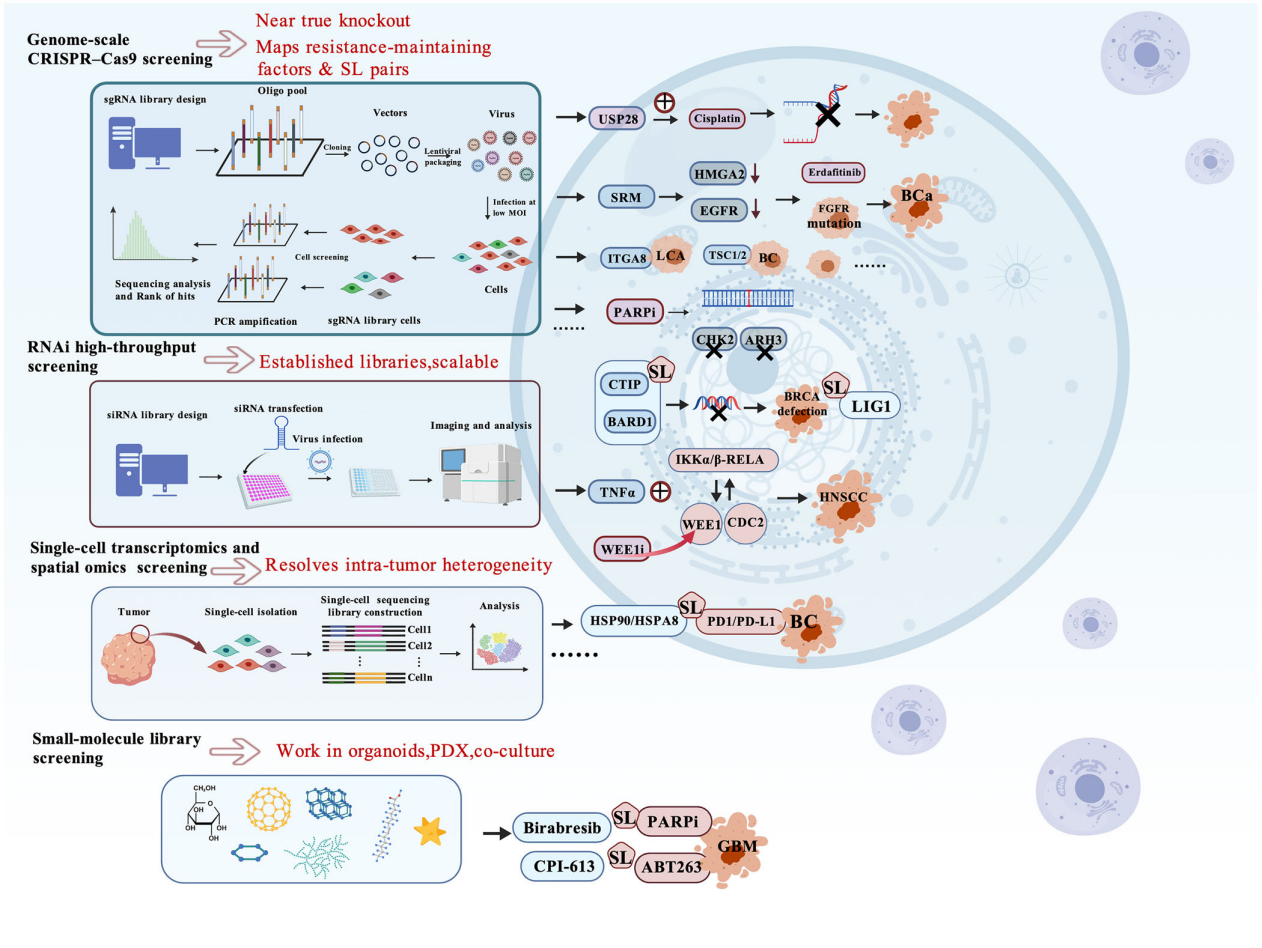
Application of synthetic lethality screening in drug resistance models. The application of synthetic lethal screening in drug resistance models can be categorised into several aspects: genome‐wide CRISPR–Cas9 screening, high‐throughput RNAi screening, single‐cell transcriptomics and utetheisa kong inter‐omics‐assisted screening and small‐molecule library screening. *Abbreviations*: USP28, ubiquitin specific peptidase 28; SRM, spermidine synthase; HMGA2, high mobility group protein A2; EGFR, epidermal growth factor receptor; BCa, bladder cancer; ITGA8, integrin subunit alpha 8; LCA, lung cancer; TSC1/2, tuberous sclerosis 1/2; BC, breast cancer; ARH3, ADP‐ribosylhydrolase 3; CTIP, C‐terminal binding protein interacting protein; BARD1, BRCA1‐associated RING domain 1; LIG1, DNA ligase 1; TNFα, tumour necrosis factor‐α; IKKα/β, inhibitor of nuclear factor kappa‐B kinase α/β; RELA, nuclear factor kappa‐light‐chain‐enhancer of activated B cells, NF‐κB; CDC2, cell division cycle 2; HNSCC, head and neck squamous cell carcinoma; HSP90, heat shock protein 90; HSPA8, heat shock protein family A member 8; GBM, glioblastoma.

Overall, these four classes of strategies are complementary in dissecting resistance‑associated SL: CRISPR/RNAi furnish a causal intervention framework; single‑cell and spatial omics reveal heterogeneity and environmental context; and small‑molecule library screens build a bridge to clinical translation. Together, they provide a solid foundation for overcoming clinical drug resistance and implementing personalised combination therapies (Figure 5).

### 4.5 Bioinformatics tools for SL prediction

In the field of bioinformatics prediction of tumour SL, a variety of representative tools and algorithms have emerged in recent years. These methods, based on distinct technical routes and data‑analytic strategies, provide important support for systematically identifying novel targets and rational drug combinations.

Statistical and multi‑omics mining methods, such as DAISY, SILI and SLIdR, rely on large‑scale cancer genomics and functional screening data. By leveraging patterns of mutual exclusivity/co‑occurrence, survival analysis and perturbation‑based viability readouts, they systematically identify SL dependencies associated with specific oncogenic drivers or tumour suppressor genes. For example, Dou et al. described a computational framework that combines machine learning and statistical inference, using Random Forest and one‑class support vector machines (One‑Class SVM) for unbiased prediction, and further incorporating mutual exclusivity and differential expression analysis to prioritise cancer‑type‐specific SL interactions, ultimately identifying 14,582 SL gene pairs across 33 cancer types.^152^ In addition, De et al.^153^ analysed genome‑wide CRISPR screening data together with molecular profiles from more than 700 cancer cell lines to identify SL interactions between paralogous genes. Owing to their data‑driven nature, these approaches can to some extent recapitulate genetic interaction patterns observed in real‑world clinical cohorts.

Knowledge graph‑ and graph neural network (GNN)‐based approaches, represented by KG4SL, MGE4SL and GCATSL, primarily integrate multiple sources of biological knowledge, including PPI, Gene Ontology (GO) annotations, pathways and disease associations, to capture putative SL interactions within complex genome‑scale networks. Recently, a SLKG was developed that integrates large‑scale entities from multiple tumour types, providing a panoramic view of SL and SDL in cancer therapy.^117^ Similarly, Hao et al. pointed out that SL data can be naturally represented as graphs, enabling GNN‑based graph learning methods to effectively model complex relationships among genes.^154^ These approaches substantially enhance the ability to uncover hidden and nonlinear interactions and are particularly valuable when direct experimental evidence for specific SL pairs is lacking, as they can predict candidate interactions from network topology.

Finally, representation learning‐based methods, exemplified by PTGNN and NSF4SL, offer more generalisable and robust predictive frameworks in scenarios where annotated SL samples are limited and true negative samples are difficult to define. As noted by Fan et al., CRISPR double‑knockout technologies can only screen hundreds of genes and combinations at a time, thus requiring efficient SL prediction models to guide experimental design.^155^ They developed a novel two‑step multi‑layer encoder for an individual‑sample‐specific SL prediction model (MLEC‑iSL), which achieved high predictive performance in K562 (AUPR 0.73; AUC 0.72) and Jurkat (AUPR 0.73; AUC 0.71) cells. Moreover, Feng et al. systematically evaluated 12 machine learning methods and found that representation learning approaches show superior cross‑dataset generalisability.^156^ By applying unsupervised or semi‑supervised learning to large amounts of unlabelled data, these methods extract general gene representations and thereby enable accurate SL prediction under conditions of sparse annotation. A concise comparison of representative tools/algorithms is summarised in Table 1.

**TABLE 1:** Representative computational tools and algorithms for synthetic lethality (SL) prediction.

Category	Tool	Core concept	Major advantages	Typical application scenarios
Statistical and multi‑omics mining methods	DAISY	Infers SL pairs from mutual exclusivity/co‑occurrence and co‑expression patterns of genomic alterations in cancer cohorts	Multi‑omics integration; good interpretability	Pan‑cancer SL network construction; SL partners of key driver genes
	SILI	Liver‑cancer‐specific pipeline combining functional similarity, differential expression, co‑expression, survival analysis and rank aggregation	Strong cancer‑type specificity; integrates molecular and prognostic information	Identification of TSG–SL pairs and network construction in hepatocellular carcinoma
	SLIdR	Rank‑based statistical framework that compares viability of mutant vs wild‑type cell lines under CRISPR/shRNA perturbations	Directly leverages functional screening data; clearer causal inference	Mining driver‑gene‐associated SL interactions from existing genome‑wide perturbation screens
Knowledge graph and graph neural network methods	KG4SL	Integrates PPI, GO, pathway and disease relationships into a knowledge graph and uses KGNN to learn gene representations for SL prediction	Exploits rich biological knowledge; suitable for genome‑wide SL prediction	Global mapping of SL/SDL landscapes and discovery of novel interactions based on large‑scale knowledge graphs
	MGE4SL	Multi‑graph ensemble; embeds PPI, GO, pathway and other networks separately and integrates them for prediction	Multi‑network fusion; high overall predictive performance	Large‑scale SL candidate prioritisation when diverse network data are available
	GCATSL	Graph context attention network introducing node‑ and feature‑level attention on GO+PPI graphs	Highlights key neighbours and features; outperforms standard GCNs	Predicting potential SL pairs from network topology when direct experimental data are scarce
Representation learning methods	PTGNN	Pretrained GNN that learns general gene representations via unsupervised graph reconstruction, then transfers them to SL prediction	Well suited to limited labelled SL data; good generalisability	Cross‑dataset or cross‑cancer SL prediction and transfer learning
	NSF4SL	Contrastive learning–based ranking framework that treats SL prediction as gene prioritisation without explicit negative labels	Avoids noisy negative labelling; suitable for ‘few positive’ settings	Prioritising candidate SL gene pairs to guide high‑cost CDKO and related experiments

## 5 INHIBITORS BASED ON SL

### 5.1 Clinically approved agents

The advent of PARPis marks a major milestone in translating SSB repair‐based SL into clinical practice, particularly for cancers harbouring BRCA1/2 mutations. This therapeutic paradigm exemplifies the strategy of exploiting intrinsic tumour defects.^14^ As core components of the base excision repair pathway, PARP enzymes repair DNA SSBs. Using NAD^+^ as a cofactor, PARP catalyses poly(ADP‑ribosyl)ation of target proteins at sites of DNA damage.^158^

The SL interaction between BRCA1/2 deficiency and PARP inhibition arises from the lethal convergence of defects in DNA repair pathways. BRCA1/2 are key regulators of HR, the principal mechanism for repairing DNA DSBs. Loss of BRCA function leads to HR deficiency (HRD).^159^ When PARPis are administered, PARP‑mediated SSB repair is effectively blocked. Unrepaired SSBs are then converted into DSBs during DNA replication. In BRCA‑mutant cells, these DSBs cannot be repaired due to HRD, resulting in replication‑fork collapse, accumulation of single‑stranded DNA gaps and ultimately catastrophic genomic instability^160^ – the molecular hallmark of SL.

Clinically, multiple PARPis have been approved for the treatment of BRCA‑mutant breast, ovarian and pancreatic cancers, validating SL as a feasible therapeutic framework. PARPis are anti‐cancer therapies targeting poly(ADP‐ribose) polymerase (PARP). As the first class of anti‐cancer drugs to gain clinical approval by exploiting the concept of SL,^161^, ^162^ they established a foundation for the development of related SL‐based therapeutics. Multiple PARPi have now been approved in China and globally. These include niraparib, pamiparib, olaparib, talazoparib and fluzoparib.

Niraparib is the first PARPi approved by the United States Food and Drug Administration (US FDA) as maintenance therapy for platinum‐sensitive OC. It acts as a PARP‐1/2 inhibitor.^163^ Pamiparib is a potent, selective, oral PARP1/2 inhibitor with a mechanism of action similar to niraparib. In preclinical studies, pamiparib demonstrated strong PARP–DNA complex trapping, efficient blood–brain barrier penetration, anti‐tumour activity and robust inhibition of PARylation.^164^ A Phase II study showed that pamiparib achieved a durable response rate (median duration of response, 11.1 months) and progression‐free survival (median PFS, 6.2 months) in patients with platinum‐resistant OC (PROC).^165^

Olaparib is used to treat cancers with specific DNA repair defects. These include cancers in patients with BRCA1 or BRCA2 mutations. Early studies showed that olaparib has fewer adverse events. It also exhibits anti‐tumour activity in cancers associated with BRCA1 or BRCA2 mutations.^166^ Both olaparib and niraparib are approved for the treatment of high‑grade epithelial OC; however, is approved as first‐line maintenance therapy following response to chemotherapy in patients carrying pathogenic BRCA mutations.^167^ In December 2024, approval was granted in mainland China for the adjuvant therapy indication of olaparib (CYHB2402053) in breast cancer,^168^ which marks the first approved targeted therapy in China for early breast cancer with BRCA mutations.

Compared with first‑generation PARPis such as olaparib, talazoparib has stronger binding affinity for PARP proteins and superior blood–brain barrier penetration. On 5 November, 2024, the National Medical Products Administration (NMPA) approved the marketing application for talazoparib tosylate capsules.^169^ Talazoparib has shown anti‐tumour activity in patients with advanced breast cancer and in those carrying germline BRCA1 and BRCA2 (BRCA1/2) mutations.^170^

In addition, several PARPis such as rucaparib and veliparib are in various stages of clinical development. Rucaparib is a PARPi with anti‐cancer activity in recurrent OC carrying BRCA mutations or with a high percentage of genome‐wide loss of heterozygosity. In a Phase III trial published in 2017, rucaparib significantly improved PFS in patients with platinum‐sensitive OC who had responded to platinum‐based chemotherapy.^171^ More recently, results from a Phase III study reported in 2025 supported rucaparib as an optimal maintenance option for patients with BRCA‑mutated recurrent OC.^172^ Emerging evidence has also revealed an interaction between PARP‑1 and the core circadian clock component BMAL1, suggesting that circadian rhythms may modulate PARPi efficacy. In rucaparib‑treated patients, expression of core clock genes (BMAL1 and PER2) differed significantly from that in the placebo group.^173^

In one study, venadaparib was identified as a novel selective PARPi with improved physicochemical properties, efficacy and safety.^174^ An ongoing Phase Ib/IIa clinical trial is currently evaluating the efficacy and safety of veliparib‑based regimens.

Key information on representative PARPi is summarised in Table 2.

**TABLE 2:** Selected approved PARP inhibitors (PARPi).

Drug	Target	Indication	Half‑life	Approval date
Niraparib	PARP1/2	Maintenance treatment for adult patients with platinum‑sensitive, recurrent epithelial ovarian, fallopian tube or primary peritoneal cancer	36 h	US FDA: 2017^175^; NMPA: 2019^176^
Pamiparib	PARP1/2	Treatment of patients with recurrent, advanced ovarian, fallopian tube or primary peritoneal cancer harbouring germline BRCA mutations who have received at least two prior lines of chemotherapy	13 h	NMPA: 2021^165^
Olaparib	PARP1/2	Treatment of cancers with specific DNA repair defects, including those harbouring BRCA1 or BRCA2 mutations.	(14.9 ± 8.2) h	US FDA: 2017; NMPA: 2018^177^
Talazoparib	PARP1/2	Patients carrying germline BRCA1 and BRCA2 (BRCA1/2) mutations	50 h	NMPA: 2024
Fluzoparib	PARP1/2	Treatment of patients with platinum‑sensitive recurrent ovarian, fallopian tube or primary peritoneal cancer harbouring germline BRCA mutations (gBRCAm) who have received at least two prior lines of chemotherapy	(9.14 ± 2.38) h	NMPA: 2020^178^
Senaparib	PARP1/2	Maintenance treatment for adult patients with advanced epithelial ovarian, fallopian tube or primary peritoneal cancer who have achieved a complete or partial response after first‑line platinum‑based chemotherapy	8.92 h	NMPA: 2025^179^

At present, most clinically available PARPis show limited selectivity between PARP1 and PARP2.^180^ Future research should prioritise the development of more precise biomarkers. These biomarkers would further optimise patient stratification and facilitate exploration of the potential therapeutic role of PARPi in earlier disease stages. The formulation of individualised treatment strategies is critical for enhancing PARPi efficacy and minimising unnecessary toxicity. Additional studies are thus essential to advance precision medicine in this context.

### 5.2 Clinical trial phase

#### 5.2.1 DNA Polθ inhibitors

DNA Polθ belongs to the family A DNA polymerases. It functions as a key DNA repair enzyme in microhomology‐mediated end joining. Studies have shown that Polθ is expressed at minimal levels in normal tissues. It is highly expressed, however, in multiple tumour types including breast, ovarian and lung cancers. These tumours often exhibit prevalent defects in HR repair. Polθ is thus considered a promising target for the development of SL‐based therapeutics.^22^

In BRCA‐deficient tumours, inhibition of Polθ can induce SL. This makes it an attractive anti‐tumour strategy in this setting. It can be used as monotherapy or in combination with PARPis. Although no Polθ inhibitor has yet received approval, several are in clinical development. For example, ART558, developed by Artios Pharma, is a Polθ inhibitor. It induces DNA damage and SL in BRCA1/BRCA2‐mutant tumour cells. It also enhances the effects of PARPi.^181^ This inhibitor elicits SL not only with BRCA gene defects but also with deficiencies in the 53BP1/Shieldin DNA repair complex.

In addition, Zhou et al. identified the compound NVB as a highly selective Polθ inhibitor through high‐throughput screening.^182^ NVB selectively kills BRCA1‐ and BRCA2‐deficient cells while exhibiting low toxicity toward wild‐type cells. Multiple Polθ inhibitors have been developed by pharmaceutical companies worldwide. They are currently undergoing clinical investigation. Examples are summarised in Table 3.

**TABLE 3:** Selected investigational Polθ inhibitors.

POLθi	Indications	Mechanism of action	Development stage	Development date
IDE705	Advanced solid tumours	Inhibits helicase activity, inducing DNA damage	Clinical trial application submitted	2023
RP‐3467	Advanced malignant solid tumours	Inhibits helicase activity, inducing DNA damage and synthetic lethality	Phase I clinical trial	2024
SIM‐0508	Advanced malignant solid tumours	Inhibits helicase activity, inducing DNA damage and synthetic lethality	Phase I clinical trial	2024
SYN‐818	Locally advanced malignant solid tumours, ovarian cancer, metastatic solid tumours	Induces DNA damage and synthetic lethality; combination with PARPi, chemoradiotherapy and antibody–drug conjugates (ADCs) to overcome resistance and reduce toxicity	Phase I clinical trial	2024
ART4215	Metastatic solid tumours, breast cancer, HER2‐negative breast cancer	Inhibits polymerase activity, inducing DNA damage and synthetic lethality; combination with PARPi/DNA‐damaging therapies	Phase I/II clinical trial	2021
ART6043	Metastatic solid tumours, advanced malignant solid tumours	Inhibits polymerase activity, inducing DNA damage and synthetic lethality	Phase I/II clinical trial	2023
GSK4524101	Solid tumours	Inhibits helicase activity, inducing DNA damage and synthetic lethality	Phase I/II clinical trial	2023

#### 5.2.2 WRN inhibitors

Werner syndrome RecQ helicase (WRN), a member of the RecQ helicase family, plays a critical role in maintaining genomic integrity and participates in DNA repair pathways such as NHEJ and HR.^183^ WRN has recently been identified as a novel SL target in MSI tumours. This supports the development of therapeutics for MSI‐driven cancers.^184^, ^185^

In 2024, Ferretti et al. used chemical screening and structure‐based design to identify HRO761, a candidate with potent WRN inhibitory activity.^186^ In MSI cells, HRO761 induces time‑ and dose‑dependent cell‑cycle arrest and DNA damage via a p53‑independent mechanism.^187^ Similarly, VVD‐133214 (RO7589831) can drive SL in MSI‐H CRC cells via covalent inhibition of WRN. This mechanism is also p53 independent.^188^ GSK4418959 is an oral allosteric WRN inhibitor that exerts SL in dMMR/MSI‐H tumours. SYLVER is an open‐label, multi‐centre, first‐in‐human, Phase 1/2 study (NCT06710847). It assesses the inhibitor's safety, tolerability, PK and pharmacodynamics in patients with advanced dMMR/MSI‐H solid tumours.^189^ A summary is provided in Table 4. Additional WRN inhibitors are currently in preclinical development and are detailed in Section 5.3.

**TABLE 4:** WRN inhibitors in clinical development.

WRNi	Indication	Mechanism of action	Development stage	Trial ID	Year initiated
VVD‐133214 (RO7589831)	Solid tumours	Allosteric inhibitor of the WRNi (4.4 ± 1.8 h)	Phase I clinical trial	NCT06004245	2024
HRO761	Colorectal cancer; solid tumours	DNA‐damaging agent; allosteric inhibitor of the WRNi	Phase I clinical trial	CTR20241049 NCT05838768	2024; 2023
GSK4418959	dMMR/MSI‑H tumours	Allosteric inhibitor of the WRNi	Phase I clinical trial	NCT06710847	2024

#### 5.2.3 PRMT5 inhibitors

PRMT5 is an epigenetic enzyme involved in multiple physiological processes. PRMT5 forms a synthetic lethal (SL) pair with MTAP.^190^, ^191^ Loss of the MTAP gene increases the dependence of MTAP‐deleted tumours on PRMT5. Inhibition of PRMT5 affects downstream cellular processes such as R‐loop formation and DNA damage. Several PRMT5 inhibitors have entered clinical evaluation.

First‐generation PRMT5 inhibitors such as GSK3326595 do not distinguish normal cells from MTAP‐deficient tumour cells. Hematologic toxicities such as anaemia limit their clinical application.^27^ In contrast, second‑generation PRMT5 inhibitors selectively bind the PRMT5–MTA complex and preferentially target MTAP‑deleted tumour cells. TNG908 is a selective, blood–brain barrier‐penetrant compound that cooperates with MTA to inhibit PRMT5. It is synthetically lethal with MTAP‐deleted cancers.^192^ TNG462 is a next‑generation second‑generation inhibitor that targets the PRMT5–MTA complex and is primarily intended for MTAP‑deleted cancers. Preclinical studies indicate that TNG462 has superior potency and selectivity compared with TNG908.^193^ Similarly, BMS‐986504 (MRTX1719) is a second‐generation inhibitor targeting PRMT5–MTA. It has been found to exhibit dose‐dependent anti‐tumour activity. It also inhibits PRMT5‐dependent SDMA modifications in MTAP‐deleted tumours.^194^ AMG193 is another second‐generation inhibitor targeting PRMT5–MTA. It was recently found to preferentially bind PRMT5 in the presence of MTA.^195^ In vitro, PRMT5 inhibition induces DNA damage, cell cycle arrest and aberrant selective mRNA splicing in cells with MTAP deletion (Table 5).^196^

**TABLE 5:** PRMT5 inhibitors in clinical development.

PRMT5i	Indication	Mechanism of action	Development stage	Trial ID
TNG462	MTAP‑deleted tumours (including non‑small cell lung cancer, melanoma, mesothelioma, gallbladder adenocarcinoma and so on	Selective binding to PRMT5–MTA complex, leading to DNA damage, cell‑cycle arrest and aberrant alternative mRNA splicing	Phase I/II clinical trials	NCT06922591; NCT06188702; NCT05732831
BMS‐986504 (MRTX1719)				NCT06672523
AMG193				CTR20250026; NCT06593522; ChiCTR2500095932

#### 5.2.4 USP1 inhibitors

Ubiquitin‑specific protease 1 (USP1) is a nuclear DUB. Inhibition of USP1 or its downstream signalling has been shown to sensitise HRD tumour cells to PARPis.^197^ Consequently, USP1 is considered a promising synthetic lethal target in HRD‑mutant tumours and may act synergistically with PARPis. ML323, the first selective USP1 inhibitor described, significantly suppresses the growth of BRCA1‑mutant OC cell lines.^198^ Additional selective USP1 inhibitors, including SIM0501,^199^ TNG348^200^ and Ksq‑4279 (RO7623066),^201^ are summarised in Table 6.

**TABLE 6:** USP1 inhibitors in clinical development.

USP1i	Indication	Mechanism of action	Development stage	Trial ID	Year initiated
SIM0501	Advanced solid tumours	Inhibits USP1 function, impairs DNA repair and enhances sensitivity to PARP inhibitors	Phase I clinical trial	NCT06331559	2024
TNG348	HRD‑associated tumours (e.g., breast, ovarian cancer)	Inhibits USP1 function, impairs DNA repair and enhances sensitivity to PARP inhibitors	Phase I/II clinical trial	NCT06065059	2023
Ksq‑4279 (RO7623066)	Solid tumours	Selective USP1 inhibition (mechanism under clinical evaluation)	Phase I clinical trial	NCT05240898	2021
SIM0501	Advanced solid tumours	Inhibits USP1 function, impairs DNA repair and enhances sensitivity to PARP inhibitors	Phase I clinical trial	NCT06331559	2024

#### 5.2.5 ATR inhibitors

Ataxia telangiectasia and Rad3‑related kinase (ATR) is a central regulator of the DDR. Cancer cells frequently suppress key DDR pathways, such as the ATM–CHK2–p53 axis.^202^ This inactivation forces tumour cells to rely on compensatory mechanisms to stabilise replication forks, among which the ATR–CHK1 pathway is pivotal.^203^

Recent studies have shown that elimusertib (BAY 1895344), a potent oral ATRi, achieves clinically meaningful disease control in patients with DDR‑deficient tumours, particularly gynaecologic malignancies.^204^ In addition, the ATRi AZD6738 (ceralasertib) displays anti‐tumour activity when combined with DNA‑damaging chemotherapeutic agents such as cisplatin.^205^ A Phase I trial in melanoma demonstrated that ceralasertib in combination with paclitaxel is generally well tolerated (Table 7).^206^

**TABLE 7:** ATR inhibitors in clinical development.

ATRi	Indication	Mechanism of action	Development stage	Trial ID	Year initiated
BAY 1895344	Advanced solid tumours; non‑Hodgkin lymphoma; treatment‑refractory solid tumours; high‑grade serous ovarian cancer; non‑small cell lung cancer	Inhibits ATR‑mediated signalling, thereby blocking activation of DNA damage checkpoints, impairing DNA repair and inducing tumour cell apoptosis	Phase I clinical trials	NCT04576091; NCT05071209	2022; 2021
AZD6738 (ceralasertib)			Phase I/II/III clinical trials	NCT06769126; NCT06732401; NCT06929260	2025

#### 5.2.6 CHK1/2 inhibitors

CHK1 and CHK2 are important components of the DDR pathway. They are activated by ATR and ATM in response to replication stress or DNA damage.^207^ Prexasertib (LY2606368) is a selective, ATP‐competitive, small‐molecule inhibitor of CHK1 and CHK2. In a Phase II clinical trial, prexasertib demonstrated clinical activity with good tolerability in patients with HGSOC harbouring BRCA‐wild‐type status.^208^

In addition, GDC‐0575 is a highly selective, oral, small‐molecule CHK1 inhibitor. In a Phase I clinical trial, GDC‐0575 was shown to be usable as monotherapy and in combination with gemcitabine, but overall tolerability in combination with gemcitabine was moderate.^209^ PHI‐101 is the first orally available CHK2 inhibitor. It has shown anti‐tumour activity in OC cell lines (Table 8).^210^

**TABLE 8:** CHK1/2 inhibitors in clinical development.

CHK1/2i	Indication	Mechanism of action	Development stage	Trial ID	Year initiated
LY2606368 (CHK1/2)	Recurrent high‑grade serous ovarian cancer	Inhibits CHK1 kinase activity, disrupting cell‑cycle control and DNA damage repair	Phase II clinical trials	NCT06597565; NCT05548296; NCT04095221	2024; 2022; 2019
GDC‑0575 (CHK1)	Lymphoma; solid tumours	Selective CHK1 inhibitor, impairs cell‑cycle regulation and DNA damage repair	Phase I clinical trial	NCT01564251	2012
PHI‑101 (CHK2)	Acute myeloid leukaemia; high‑grade serous ovarian cancer	Inhibits CHK2 activity, affecting cell‑cycle regulation and DNA damage repair	Phase I clinical trials	NCT04678102; NCT04842370	2020

#### 5.2.7 Other inhibitors

The BAF complex (the major subcomplex of SWI/SNF) is fully dependent on SMARCA2 for its function. Inhibition of SMARCA2 leads to complete inactivation of the BAF complex, transcriptional failure and ultimately cell death, constituting a synthetic lethal interaction. In NSCLC, SMARCA4 mutations occur in up to 11% of cases, and notably, 84.2% of affected patients already present with distant metastases at diagnosis.^211^ SMARCA4 mutations or deletions have also been reported in other malignancies, including OC, melanoma and NSCLC.^212^

PRT3789 is the first PROTAC degrader targeting SMARCA2 to enter clinical testing. It recruits an E3 ligase to induce highly selective (>1000‑fold) degradation of SMARCA2 and demonstrates anti‐proliferative activity against SMARCA4‑deficient tumours in vitro and in vivo with good tolerability.^213^ A947 is another potent, moderately selective SMARCA2 degrader, which achieves approximately 28‑fold preferential degradation of SMARCA2 with minimal growth inhibition in SMARCA4‑wild‑type tumours. A947 (PRT7732) entered a Phase I clinical trial in August 2024.^214^ Additional inhibitors remain in preclinical development and are discussed in Section 5.3.

PLK4 is a key kinase regulating centrosome duplication. In TRIM37‑amplified tumours (such as neuroblastoma and breast cancer), PLK4 inhibition induces centrosomal dysfunction and mitotic abnormalities, ultimately leading to cell death—a synthetic lethal effect. CFI‑400945 is a PLK4 inhibitor being evaluated in Phase II trials for the treatment of acute myeloid leukaemia and chronic myelomonocytic leukaemia, as well as solid tumours.^215^ In parallel, RP‑1664, a novel PLK4 inhibitor, disrupts centriole biogenesis and is effective in TRIM37‑amplified tumours. RP‑1664 has entered a Phase I trial (NCT06232408) for patients with advanced solid tumours.^18^

RAD51, the central recombinase of the HR pathway, is essential for both cell‑cycle progression and DNA damage repair. BRCA1/2 mutations ultimately result in HR deficiency in part because of an inability to form RAD51 nucleofilaments. Therefore, pharmacological inhibition of RAD51, particularly in combination with PARP inhibition, can induce SL.^216^ Recent work has identified a new class of BRCA2–RAD51 PPI inhibitors, exemplified by a dihydroquinolinyl‑pyrazine compound (compound 35d), which disrupts the RAD51–BRCA2 interaction and phenocopies BRCA2 loss. This program remains at the research stage.^217^

Amplification of CCNE1 leads to hyperactivation of cyclin E–CDK2, driving high levels of replication stress and DNA damage and rendering tumour cells highly dependent on the G2/M checkpoint.^218^ PKMYT1 delays mitotic entry by inhibiting CDK1. In CCNE1‑amplified cells, dose‑dependent inhibition of PKMYT1 causes premature CDK1 activation and G2/M checkpoint collapse, thereby inducing a dose‑dependent synthetic lethal effect.^219^ RP‑6306 is a selective PKMYT1 inhibitor that suppresses the growth of CCNE1‑amplified cells and tumours.^220^ A first‑in‑human trial of RP‑6306 is ongoing as monotherapy (NCT04855656) and in combination with gemcitabine (NCT05147272) or FOLFIRI (NCT05147350). Additional PKMYT1 inhibitors, including compound 8 m^221^ compound A30^222^ and compound 7,^223^ are currently at the preclinical stage and are summarised in Section 5.3.

The detailed information on these inhibitors is summarised in Table 9.

**TABLE 9:** Other synthetic lethal inhibitors in clinical development.

Target	Compound / drug	Indication	Mechanism of action	Development stage	Trial ID	Year initiated
SMARCA2/4 inhibitors	A947	SMARCA4‑deficient tumours such as non‑small cell lung cancer, colorectal cancer, etc.	Recruits VHL to induce SMARCA2 ubiquitination and subsequent proteasomal degradation, exerting anti‐tumour activity	Phase I clinical trial	NCT06560645	2024
	PRT3789			Phase I/II clinical trials	NCT06682806; NCT05639751	2025; 2023
PLK4 inhibitors	CFI‑400945	Advanced triple‑negative breast cancer; metastatic breast cancer; prostate adenocarcinoma	Inhibits PLK4 kinase activity, disrupts cell‑cycle progression in cancer cells, thereby inducing apoptosis or growth inhibition	Phase II clinical trials	NCT04730258; NCT04176848; NCT03624543	2021; 2020; 2019
	RP‑1664	Breast cancer; neuroblastoma; non‑small cell lung cancer		Phase I clinical trial	NCT06232408	2024
PKMYT1 inhibitors	RP‐6306	Advanced solid tumour	Inhibiting PKMYT1 kinase activity, interfering with cell cycle regulation and inducing cancer cell death	Phase I clinical trial	NCT04855656	2021

### 5.3 Preclinical stage

Recent work has identified ZM‑3329 as a novel, potent WRN inhibitor that induces accumulation of DNA damage signatures in MSI‑H cells by targeting WRN. ZM‑3329 exhibits robust anti‐tumour activity in xenograft models derived from multiple tissue types as well as in PDOs.^224^ In addition, MOMA‑341 is a new, potent and selective small‑molecule WRN inhibitor in clinical‑stage development that induces DNA damage, cell death and tumour regression in dMMR/MSI‑H models. Notably, MOMA‑341 is associated with a more refined functional predictive biomarker – TA repeat expansions – which can almost perfectly predict sensitivity to MOMA‑341 across diverse MSI‑H tumour models, supporting highly precise patient selection.^225^

PROTACs represent an emerging drug‑discovery strategy that suppresses disease progression by promoting targeted protein degradation rather than simple enzymatic inhibition. Several SMARCA2/4‑directed degraders are currently in preclinical development. For example, ACBI2 (CAS: 2913161‑19‑8) robustly degrades SMARCA2 in human whole blood from three healthy donors ex vivo, with clear selectivity over SMARCA4, as shown by Kofink and colleagues. SMD‑3040 is a highly efficient and selective SMARCA2 degrader (>80‑fold selectivity) with preferential activity in SMARCA4‑deficient tumour cells. It shows excellent tumour tissue penetration, with markedly slower clearance in tumours than in plasma.^226^ Simcere has reported the SMARCA2 degrader ZM‑0011 (SCR‑9140), which significantly reduces intra‐tumoural SMARCA2 levels and suppresses tumour growth in mouse models, with a favourable tolerability profile.^227^

PKMYT1 is a serine/threonine kinase responsible for the inhibitory phosphorylation of CDK1 and plays a central role in regulating the G2/M cell‑cycle checkpoint. Most reported PKMYT1 inhibitors to date are structural analogues of RP‑6306. Recent studies have identified several promising candidates, including compound A30, compound 7 and compound 8 m, as potent PKMYT1 inhibitors.

Zhang et al.^222^ reported that compound A30 displays outstanding PKMYT1 kinase inhibitory activity ((IC50) = 0.003 µM) and exerts strong anti‐proliferative effects in CCNE1‑amplified tumour cells. Similarly, Lu and colleagues discovered a series of 2‑amino‑[1,1′‑biphenyl]‑3‑carboxamide derivatives – exemplified by RE3 (compound 8ma) – as PKMYT1 inhibitors, in which the aminopyrrolidine moiety provides additional stabilising hydrogen‑bond interactions.^221^ Moreover, compound 7, a pyrrolopyrimidinone derivative, has emerged as a potent PKMYT1 inhibitor that strongly suppresses PKMYT1 and pCDK1, while exhibiting favourable metabolic stability (Table 10).^223^

**TABLE 10:** Synthetic lethal inhibitors in preclinical development.

Inhibitor class	Drug/compound	Indication	Mechanism of action	Development stage
WRN inhibitors	ZM‐3329	Solid tumours	Apoptosis inducer; DNA‐damaging agent; allosteric inhibitor of WRN	Preclinical
	MOMA‐341	Endometrial, gastric, colorectal and other solid tumours	Allosteric inhibitor of Werner syndrome ATP‐dependent helicase (WRN)	Preclinical
SMARCA2/4 degraders	ACBI2	SMARCA4‐deficient tumours such as non‐small cell lung cancer, colorectal cancer and so on	PROTAC that recruits the VHL E3 ubiquitin ligase, inducing SMARCA2 ubiquitination and proteasomal degradation, leading to anti‐tumour activity	Preclinical
	SMD‐3040			Preclinical
	ZM‐0011 (SCR‐9140)			Preclinical
PKMYT1 inhibitors	Compound A30	CCNE1‐amplified tumours such as breast and ovarian cancer	Disrupts G2/M checkpoint control, leading to CDK1 hyperactivation and accumulation of DNA damage	Preclinical
	Compound 7			Preclinical
	Compound 8 m			Preclinical

### 5.4 Emerging targets

RAD51 is the central recombinase of the HR pathway and plays a key role in the repair of DNA DSBs and in the response to replication stress. RAD51 overexpression has been observed across multiple tumour types.

Multiple small molecules have been reported that inhibit HR by directly blocking RAD51 activity or disrupting its interaction with BRCA2, thereby preventing RAD51 filament assembly and inducing synthetic‑lethal cell death in tumours with heightened dependence on HR. Examples include B02 and its analogue B02 isomer B02‑iso,^228^ which sensitise TNBC MDA‑MB‑231 cells to the PARPi olaparib, as well as the RAD51 inhibitor Cpd‑4 and others.^229^ In addition, the formation of RAD51 foci has been linked to resistance to PARPis in breast cancer patients carrying germline BRCA mutations. Consequently, RAD51 is emerging as a promising therapeutic target to restore SL in tumours that have acquired resistance to PARPis. At present, RAD51‑directed inhibitors are largely confined to the preclinical stage.

Growing attention has also focused on the RAD51 paralog family members (RAD51B, RAD51C, RAD51D, XRCC2 and XRCC3) as synthetic lethal nodes associated with BRCA deficiency. Multiple studies have shown that^230^, ^231^ pathogenic or likely pathogenic variants in these genes can compromise HR function. Patients harbouring mutations in RAD51 paralogs appear to derive clinical benefit from PARPi therapy similar to that observed in BRCA/PALB2‑mutant tumours.

As previously mentioned, amplification of the CCNE1 gene leads to overactivation of the CCNE1‐CDK2 complex, thereby inducing high levels of replication stress and DNA damage. In CCNE1‐amplified cells, dose‐dependent inhibition of PKMYT1 triggers premature activation of CDK1 and collapse of the G2/M checkpoint, resulting in a dose‐dependent synthetic lethal effect. Recent studies have found that CCNE1 overexpression can cause replication fork stalling and collapse, increasing dependence on RAD52.^232^ RAD52 stabilises stalled replication forks by binding single‐stranded DNA and promoting strand annealing.^233^ Through break‐induced replication (BIR), RAD52 mediates the processing and restart of replication forks stalled due to DNA replication stress. Research has identified a BIR‐like mechanism called ‘mitotic DNA synthesis’ (MiDAS), which is active during early mitosis, where MUS81 is recruited to under‐replicated DNA sites to promote POLD3‐dependent DNA synthesis.^234^ In this process, RAD52 is essential for the timely recruitment of MUS81 to early mitotic under‐replicated DNA sites.^235^ Further studies on this issue revealed that mitotic degradation of RAD52 eliminates CCNE1‐driven MiDAS and reduces the survival rate of CCNE1‐overexpressing cells, highlighting the critical role of RAD52 in maintaining genomic integrity during mitosis.^229^ Therefore, inhibiting MiDAS may be an effective strategy for targeting tumours with high CCNE1 levels. Collectively, RAD51 paralogs and related factors are gradually being incorporated into a broader definition of HRD.

c‐MYC (MYC) oncoprotein is dysregulated in the majority of human cancers. As a master regulator of gene transcription, MYC controls multiple biological processes and, when deregulated, drives many of the hallmarks of cancer.^236^ Synthetic lethal vulnerabilities in MYC‑deregulated tumours describe a state in which MYC‑transformed cancer cells become dependent on specific proteins or pathways for survival.^157^ Exploiting these MYC‑driven liabilities represents a promising indirect strategy to target MYC in cancer.

Recently, Lin et al.^121^ reported that, in MYC‑driven tumours, inhibition of topoisomerase I (TOP1) promotes aberrant R‑loop accumulation, markedly exacerbates pre‑existing transcription–replication conflicts and DNA damage, and thereby induces a MYC‑selective synthetic lethal effect. Although clinical data using MYC status as a biomarker to guide TOP1 inhibitor therapy are still limited, preclinical evidence supports that MYC‑overexpressing tumours display increased sensitivity to TOP1 inhibitors.

**FIGURE 6:**
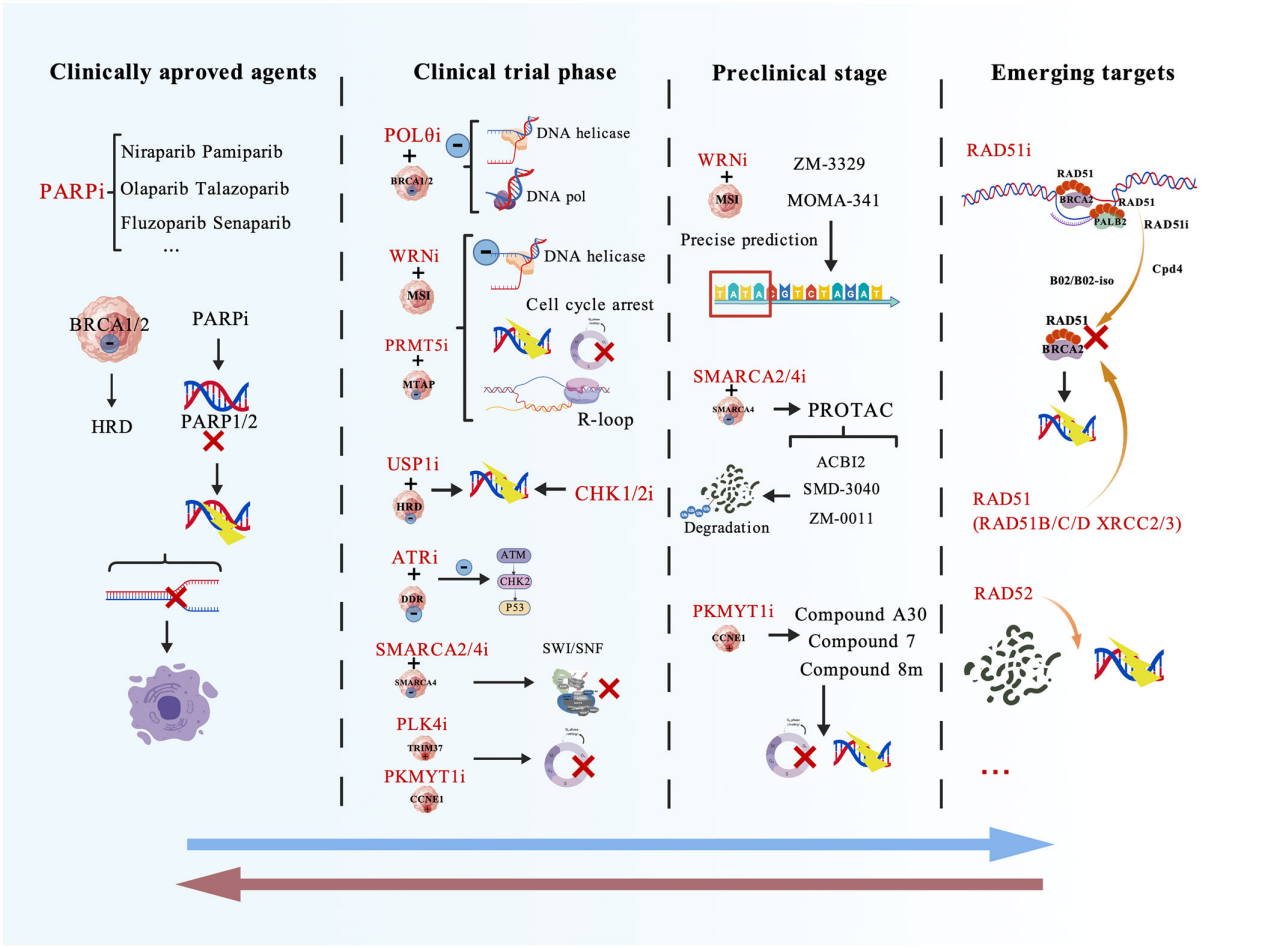
Inhibitors based on synthetic lethality. Inhibitors based on synthetic lethality can be categorised into several aspects: approved and marketed, clinical trial phase, preclinical research and emerging targets. *Abbreviations*: HRD, HR deficiency; POLθi, DNA polymerase theta inhibitor; WRNi, Werner syndrome RecQ‐Like helicase inhibitor; PRMT5i, protein arginine methyltransferase 5 inhibitor; USP1i, ubiquitin‐specific protease 1 inhibitor; ATRi, ataxia telangiectasia and Rad3‐related inhibitor; SMARCA2/4i, SWI/SNF‐related matrix‐associated actin‐dependent regulator of chromatin, subfamily A, member 2/4 inhibitor; SWI/SNF, Switch/Sucrose non‐fermentable; PLK4i, polo‐like kinase 4 Inhibitor; PKMYT1i, membrane‐associated tyrosine/threonine kinase 1 inhibitor; CCNE1, cyclin E1; RAD51i, recombination repair protein RAD51 inhibitor; Cpd4, compound 4; RAD52, recombination repair protein RAD52; RAD51B/C/D, recombination repair protein RAD51B/C/D; XRCC2/3, X‐ray repair cross complementing 2/3.

Compared with conventional strategies that directly or indirectly inhibit MYC, this approach does not require MYC itself to be druggable but instead leverages the genomic instability and vulnerabilities elicited by MYC deregulation. In addition, it can be implemented using already approved TOP1 inhibitors, shortening the translational path. Finally, this strategy can be readily combined with DDR inhibitors to establish multi‑layered synthetic lethal interactions, offering substantial potential for therapeutic enhancement (Figure 6).

## 6 SYNTHETIC LETHAL BIOMARKERS AND COMBINATION THERAPY

In SL research, biomarker discovery facilitates early disease diagnosis and rational patient selection. Based on the key signalling pathways and functional modules involved, currently studied synthetic lethal–related biomarkers can be broadly classified into the following categories: DDR and repair; cell‑cycle and mitotic regulation; translation and tRNA metabolism; regulators of cell survival and apoptosis; and metabolism‑related biomarkers.

### 6.1 Biomarkers

#### 6.1.1 DDR and repair‐related biomarkers

BRCA1 and BRCA2 are core tumour suppressor genes in the HRR pathway. Germline or somatic mutations in these genes impair HRR function and fundamentally alter how cells repair DNA DSBs.^160^ Tumours harbouring BRCA1/2 mutations are highly sensitive to PARPis, and BRCA1/2 deficiency has become one of the most robust and clinically validated predictive biomarkers for SL to date.^14^

Beyond BRCA, several factors have been associated with PARPi sensitivity, including Schlafen 11 (SLFN11),^237^ the metabolic enzymes isocitrate dehydrogenase 1 and 2 (IDH1 and IDH2)^238^, ^239^ and RAD51.^240^, ^241^ The anti‐tumour activity of PARPis derives not only from catalytic inhibition but also from ‘PARP trapping’, that is, the stabilisation of PARP–DNA complexes. The expression and functional status of SSB repair factors such as XRCC1 and LIG3 can influence the extent of PARP trapping and thus drug sensitivity.^242^, ^243^ This suggests that functional or composite biomarkers built around PARP‑trapping‐related molecules may better capture the probability of synthetic lethal responses than single‑gene mutations alone, although such markers remain under active investigation.

ATM is a key kinase in the DDR network. Loss of ATM function enhances tumour dependence on ATR signalling; therefore, ATM mutation or deletion is considered a potential predictive biomarker for ATRis. Clinical studies support synthetic‑lethal sensitivity to ATR inhibition in ATM‑deficient tumours.^114^ For example, Mei‑PROTACs‑9b was designed to induce ATM degradation and synergistically augment the activity of the ATRi AZD6738 (ceralasertib). This work established a synthetic lethal interaction between ATR inhibition and ATM deficiency, offering a novel therapeutic avenue for CRC. However, ATM status alone may be insufficient; more reliable biomarker models will likely require integrated assessment with other DDR or cell‑cycle regulators.

#### 6.1.2 Cell‑cycle and mitotic regulation‐related biomarkers

SL is not confined to DNA repair; aberrant cell‑cycle control can also expose exploitable vulnerabilities, particularly in the context of small‑molecule inhibitors targeting CDKs, CHK1/WEE1 and PLK1. CDKs coordinate DNA replication, transcription and repair by regulating cell‑cycle progression and transcriptional programs.

Prindle et al.^244^ found that interactions between the PELO‐HBS1L and SKI complexes alter the normal cell cycle and inhibit tumour growth by activating IRE1 to drive the unfolded protein response. The SL of the PELO‐HBS1L and SKI complexes can thus act as a biomarker to identify tumour patients likely to be sensitive to related therapies. Recently, Chen et al.^245^ found that the gene encoding CDP‐diacylglycerol synthase 2 (CDS2), a key enzyme in the cell‐membrane phospholipid synthesis pathway, exhibits a synthetic lethal effect in mesenchymal‐like cancers with low expression of the gene encoding CDS1. CDS2 knockout disrupts phosphatidylinositol synthesis and increases apoptosis, while re‑expression of CDS1 can rescue this adaptive defect. Thus, the CDS1/CDS2 axis may represent a potential therapeutic target across multiple cancer types.

In prostate cancer, CDK12 loss shows synergy with AKT pathway inhibition, leading to cell‑cycle arrest and apoptosis.^246^ Clinical trials are ongoing to evaluate CDK12 inhibitors in combination with AKT inhibitors, and CDK12 expression or mutational status may serve as a potential biomarker for such regimens.

PLK4 regulates centriole biogenesis. In TRIM37‑amplified tumours, such as breast cancer,^247^ PLK4 inhibition causes centrosomal dysfunction and mitotic abnormalities, resulting in cell death. PLK4 inhibitors (CFI‑400945 and RP‑1664) are currently being evaluated in clinical trials, and TRIM37 amplification is being explored as a predictive biomarker for response.

#### 6.1.3 Translation and tRNA metabolism‐related biomarkers

Emerging evidence indicates that the protein translation machinery – particularly tRNA metabolism and tRNA‑modifying enzymes – can also constitute critical nodes within synthetic lethal networks. tRNA degradation is a key factor in generating small non‐coding RNAs (tsRNAs).^248^ With advances in sequencing technologies, the number of newly discovered tsRNAs continues to grow. Recent studies indicate that tsRNAs regulate drug resistance in multiple tumours and hold promise as novel predictive biomarkers.^249^

Cui et al.^250^ found that upregulation of tDR‐0009 and tDR‐7336 in TNBC cells maintains interleukin‐6 responsiveness and induces MDR through modulation of STAT3 phosphorylation and activation. Similarly, tRF‐30‐JZOYJE22RR33 and tRF‐27‐ZDXPHO53KSN induce trastuzumab resistance in HER2‐positive breast cancer cells and can serve as new predictive biomarkers for trastuzumab‐resistant breast cancer.^251^, ^252^ In MM, Xu et al.^253^ identified through RNA sequencing and bioinformatic analyses that tRF‐60:77‐Thr‐TGT‐1 and tRF‐1:22‐Lys‐TTT‐1‐M3 may serve as clinical biomarkers of resistance and prognosis in relapsed or refractory MM, with potential as future therapeutic targets. In hormone receptor‐positive (HR^+^) breast cancer, tamoxifen resistance remains a clinical challenge. Sun et al.^254^ found that exosomal tRF‐16‐K8J7K1B targets tumour necrosis factor‐related apoptosis‐inducing ligand in recipient cells, down‐regulates expression of apoptosis‐related proteins such as caspase‐3 and poly(ADP‐ribose) polymerase, and thereby reduces drug‐induced apoptosis. Exosomal tRF‐16‐K8J7K1B may thus be a novel therapeutic target to overcome tamoxifen resistance in HR+ breast cancer. tRF‐Leu‐CAG is a recently identified tRNA‐derived small non‐coding single‐stranded RNA. Studies have shown that tRF‐Leu‐CAG promotes NSCLC tumour growth and metastasis by targeting TCEA3 and enhances paclitaxel resistance by augmenting cellular autophagy, providing potential effective targets and therapeutic options for clinical NSCLC treatment.^255^

#### 6.1.4 Metabolic and epigenetic biomarkers

ME and epigenetic remodelling are fundamental to tumour survival and adaptation to the microenvironment and also create new vulnerabilities for synthetic lethal strategies.

MTAP, located at 9p21, is frequently co‑deleted with CDKN2A/B. MTAP‑deficient tumours cannot efficiently catabolise MTA, leading to intracellular MTA accumulation and partial inhibition of PRMT5. As a result, such tumours show heightened dependence on residual PRMT5 activity and are particularly sensitive to PRMT5 inhibitors.^256^ MTAP loss is therefore emerging as a prototypical metabolic synthetic lethal biomarker for guiding the use of PRMT5‑MTA‐directed inhibitors.

Alterations in epigenetic regulators can similarly create synthetic lethal vulnerabilities at the chromatin level. The SWI/SNF chromatin complex consists of multiple subunits, with ARID1A being a key component that is frequently inactivated in cancer. leading to widespread changes in chromatin accessibility, DDRs and transcriptional regulation. Numerous studies have demonstrated increased dependence on ATR signalling in ARID1A‑deficient tumours, along with synthetic‑lethal sensitivity to ATRis.^94^ Some models also suggest that ARID1A loss may enhance responsiveness to c‑MET inhibitors, indicating potential biomarker value for DDR‑targeted therapies and selected receptor TKIs, although clinical evidence remains limited.^257^

P300/CBP are epigenetic regulators with HAT activity. In PTEN‑deficient CRC cells, P300/CBP inhibitors reduce HSP70 expression, impairing the cellular capacity to cope with stress and maintain proteostasis, and ultimately triggering synthetic‑lethal cell death.^258^ Thus, PTEN loss – classically associated with aberrant PI3K/AKT signalling – is also being explored as an epigenetic biomarker predictive of sensitivity to P300/CBP inhibition.

### 6.2 Combination therapy

A major limitation of both conventional and next‑generation single‑agent targeted therapies is the frequent emergence of resistant tumour cell subclones. Because pharmacologic synergy is highly context‑dependent, rational combinations of targeted agents can produce pronounced synergistic effects. Combination regimens not only help overcome drug resistance but can also open new therapeutic avenues.^259^ Examples include the identification of the EGFR–EPHA2 complex as a resistance node by El Zawily et al., leading to the development of the bispecific antibody BMX‑661, which showed promising activity in aggressive TNBC and pancreatic cancer; and triplet regimens used in BRAF‑mutant CRC patients.^260^ Objectives in designing combination regimens include synergistic enhancement, reduction of resistance emergence, coverage of tumour cell heterogeneity and mitigation of drug toxicity.

Accumulating evidence suggests that BRCA1/2 status alone is insufficient to fully explain clinical benefit from PARP inhibition. In BRCA‑deficient cells that acquire PARPi resistance, defects in HR, replication fork protection and gap filling are often partially or fully restored. In addition, BRCA‑deficient but PARPi‑resistant cells may increasingly rely on alternative DSB repair pathways for survival. Accordingly, combining PARPis with other DDR‑targeted agents is a rational approach to overcoming PARPi resistance—for example, PARPi with ATR/Chk1 inhibitors,^261^ PARPi with WEE1/PKMYT1 inhibitors^220^, ^262^ or PARPi with inhibitors of post‑replicative gap repair.^263^

WEE1 is a key kinase involved in the DDR pathway. Studies have found that combination treatment with MK‐1775 (a WEE1 and PLK1 inhibitor) and irinotecan (a TOP1 inhibitor) achieved a 26.7% synergy rate in microsatellite‐stable (MSS) colon cancer and 6.7% in MSI disease. Trials are currently underway in rhabdomyosarcoma and medulloblastoma (NCT02095132).^264^ WEE1 inhibitors can also be combined with PARPis to enhance tumour cell cytotoxicity. Research has shown that PARPis combined with a WEE1 inhibitor (WEE1i) target the replication stress response to enhance anti‐tumour activity and overcome PARPi resistance.^262^

For pancreatic ductal adenocarcinoma (PDAC), DNA‐damaging agents such as platinum compounds and PARPi benefit treatment but resistance arises. Studies indicate that combining the ATRi ceralasertib with the PARPi olaparib demonstrates synergy in acquired‐resistance models, providing new insights for PDAC therapy.^265^ As noted, inhibitors targeting PRMT5‐MTA exhibit SL in MTAP‐deleted tumours. Multiple PRMT5 inhibitors, including AMG193 and TNG462, are in clinical trials for treating patients with MTAP‐deleted solid tumours.

Some SL agents, while killing tumour cells, may alter the tumour microenvironment to make tumour cells more readily recognised and attacked by the immune system, thereby enhancing immunotherapy efficacy. For example, a recent study evaluated the efficacy signal of durvalumab plus ceralasertib in advanced or metastatic NSCLC after failure of prior anti‐PD‐1/PD‐L1 immunotherapy and platinum doublet therapy.^266^ Surprisingly, this treatment demonstrated significant efficacy in certain patients carrying ATM gene mutations and those with biomarker mismatch, and these cohorts exhibited both primary and acquired resistance across subgroups of patients intolerant to ICB. Durvalumab plus ceralasertib may thus become a new option after resistance to immunotherapy and platinum doublet therapy in NSCLC. Overall, because most monotherapies eventually select for resistant clones, combination therapy is likely to dominate future treatment paradigms. Advancing rational combinations will support the development of precision oncology – enabling individualised, biomarker‑driven regimens – and will also accelerate drug development and broaden clinical application.

## 7 CONCLUSIONS

SL provides a powerful conceptual framework and therapeutic strategy for tackling drug resistance in cancer. The paradigmatic example of BRCA1/2 deficiency and PARP inhibition illustrates how exploiting tumour‑specific DDR defects can achieve selective tumour cell killing without substantially increasing toxicity to normal tissues. More recently, work on DDR network reprogramming, metabolic rewiring and dependence on cell‑cycle checkpoints has extended the use of SL to reversing resistance to chemotherapy, targeted therapy and immunotherapy. An expanding repertoire of SL targets – including POLθ, WRN, the PRMT5–MTA complex, USP1 and ATR/CHK1/WEE1 – and their inhibitors is creating multi‐layered opportunities to overcome resistance across distinct molecular subtypes and disease stages.

Despite this progress, SL research and clinical translation remain uneven. First, SL interactions that have been robustly validated across multiple cohorts and platforms and moved into clinical development are still heavily concentrated in DDR pathways and a small set of metabolic targets. Truly cross‑tumour, reproducible and druggable ‘core SL modules’ are scarce. Many candidates from CRISPR/RNAi screens, small‑molecule libraries or resistance models are restricted to single cell lines or narrow genetic contexts, and their effects are often attenuated in heterogeneous real‑world populations.

Second, major screening platforms – genome‑scale CRISPR–Cas9 and RNAi libraries, small‑molecule screens and single‑cell or spatial omics – typically solve specific ‘local problems’ in SL discovery. A unifying framework and rigorous prioritisation criteria for translating high‑throughput hits into actionable clinical decisions are still lacking, forming a key bottleneck in the bench‑to‑bedside continuum. Pharmacologically, CRISPR/RNAi screens model near‑complete gene loss, whereas clinical inhibitors usually produce partial, reversible and time‑dependent target suppression. Together with on‑target mimicry and off‑target effects, this discrepancy can generate ‘pseudo‑SL’ signals or context‑dependent antagonism, complicating target selection. Experimentally, although organoids, PDX models and 3D co‑culture systems better approximate human tumours than 2D monolayers, most still lack a fully functional immune microenvironment and systemic metabolic interactions, leading to underestimation of SL interactions that depend on immune or stromal amplification.

Future efforts should shift from empirically cataloguing isolated SL pairs toward data‑driven modelling of SL networks and modules. With the maturation of resources such as TCGA, DepMap, Project SCORE and knowledge graphs like SLKG, AI‑ and machine‑learning‐based prediction of SL interactions has started to show promise. Recent studies have integrated patient multi‑omics data, genome‑wide CRISPR double‑knockout screens and graph‑based models – including GNNs, contrastive learning and pretrained representation learning – to enable context‑ or patient‑specific SL prediction. Some have further incorporated longitudinal ctDNA or single‑cell data to dynamically model SL dependencies and resistance trajectories, aiming to anticipate future resistant clones and design adaptive combination regimens. Nevertheless, these models continue to face biased training data, incomplete and noisy labels, limited cross‑cohort generalisability and ‘black‑box’ decision processes that hinder clinical adoption.

From a translational standpoint, the priority is no longer to simply ‘discover more pairs’, but to build a robust loop of mechanism–prediction–validation–application around a limited set of rigorously validated SL modules: confirming their association with resistance and prognosis in large cohorts and real‑world datasets; optimising drug combinations, dosing and scheduling in patient‑derived models; and using dynamic biomarkers – such as ctDNA, cfRNA, metabolomic signatures and immune profiling – to track SL dependencies and resistance evolution. Constructing such an iterative loop, while explicitly accounting for pharmacologic off‑target effects and model limitations, will be essential for achieving truly dynamic, resistance‑aware precision oncology based on SL.

## AUTHOR CONTRIBUTIONS

All authors contributed to the study conception and design. The first draft of the manuscript was written by Junyan Li and all authors commented on previous versions of the manuscript. All authors read and approved the final manuscript.

## CONFLICT OF INTEREST STATEMENT

The authors declare no conflicts of interest.

## CONSENT

Informed consent was obtained from all individual participants included in the study. The authors affirm that human research participants provided informed consent for publication of the images in Figures 1, 2, 3, 4, 5 and 6.

## Data Availability

The data referenced in this study are primarily sourced from publicly accessible databases: PubMed and China National Knowledge Infrastructure (CNKI). Specific literature and datasets can be accessed as follows: PubMed: A doi has been provided after each reference. China National Knowledge Infrastructure (CNKI): The Chinese literature has been translated into English, and links have been placed after the references. As this study did not generate new datasets or raw data, there are no additional data to share. All necessary data and analysis results are described in detail within the article and can be accessed through the aforementioned links.
